# Biomass-derived hydrochar and activated carbon in pharmaceutical pollution mitigation: a comprehensive overview

**DOI:** 10.1039/d5ra04857e

**Published:** 2025-11-05

**Authors:** Thi Mai Vu, Thi Minh Phuong Nguyen, Huu-Tap Van, Ngoc Thuan Le, Dinh-Trinh Tran

**Affiliations:** a Environment Faculty, Hanoi University of Natural Resources and Environment 41A, Phu Dien Road, Phu Dien Ward Hanoi city Vietnam; b Faculty of Environmental and Natural Sciences, Duy Tan University Da Nang 550000 Vietnam; c Institute of Research and Development, Duy Tan University Da Nang 550000 Vietnam; d Center for Advanced Technology Development, Thai Nguyen University Tan Thinh Ward Thai Nguyen City Vietnam tapvh@tnus.edu.vn vanhuutap@tnu.edu.vn; e Research Istitute for Resources and Climate Change, Hanoi University of Natural Resources and Environment 41A, Phu Dien Road, Phu Dien Ward Hanoi city Vietnam; f VNU Key Lab. of Advanced Materials for Green Growth, University of Science, Vietnam National University No. 19 Le Thanh Tong Street, Hoan Kiem 120000 Hanoi Vietnam

## Abstract

Hydrochar, a carbonaceous material produced from biomass *via* hydrothermal carbonization (HTC), has emerged as a sustainable adsorbent for mitigating pharmaceutical pollution in wastewater. Unlike pyrochar and hydrochar-derived activated carbon (HDAC), hydrochar is synthesized at lower temperatures (180–250 °C) using wet biomass, reducing energy consumption and enabling valorization of high-moisture waste without costly drying processes. Its rich oxygenated functional groups and tunable surface chemistry enhance the adsorption of polar contaminants through mechanisms such as hydrogen bonding, π–π interactions and hydrophobic effects, offering advantages over the more aromatic, less functionalized pyrochar. Hydrochar and HDAC demonstrate significant potential for removing pharmaceutical pollutants, with enhanced performance achievable through tailored preparation and activation methods that optimize their surface properties. Hydrochar derived from horse manure exhibits a low adsorption capacity of 1.8 μg g^−1^ for ciprofloxacin, attributed to its limited specific surface area (SSA) of 4.62 m^2^ g^−1^. In contrast, ZnCl_2_-activated HDAC with an SSA of 1326 m^2^ g^−1^ achieves a significantly higher capacity of 416.7 mg g^−1^, driven by π–π interactions and chemisorption. Similarly, KOH activation of grape seed-derived hydrochar enhances the capacity to 650.8 mg g^−1^ for sulfamethoxazole. Despite these advancements, challenges persist, including non-selective adsorption, pH sensitivity (optimal range of 6–8) and limited regeneration efficiency, with a capacity reduction of 18–23% after five cycles. Despite its potential, challenges such as non-selective adsorption, pH sensitivity and limited regeneration efficiency remain. This review highlights hydrochar and HDAC's versatility and sustainability, advocating for further research to refine activation methodologies, optimize regeneration techniques and scale its application in pharmaceutical wastewater treatment. By incorporating hydrochar into sustainable wastewater management frameworks, it is feasible to mitigate the environmental impact of pharmaceutical pollution effectively.

## Abstract 2

Hydrochar, a carbonaceous material produced from biomass *via* hydrothermal carbonization (HTC), has emerged as a sustainable adsorbent for mitigating pharmaceutical pollution in wastewater.

## Graphical abstract

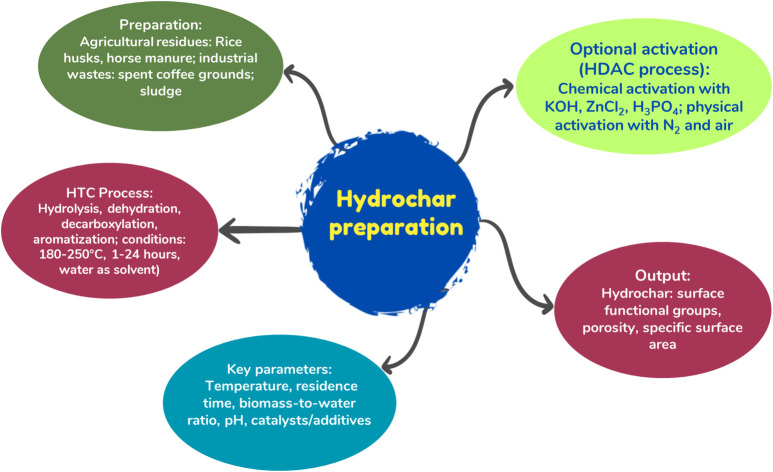

## 1. Introduction

Pharmaceuticals, including antibiotics, are emerging contaminants impacting wastewater, surface water, groundwater and drinking water, with concentrations ranging from ng L^−1^ to mg L^−1^.^1,2^ They pose a significant pollution risk compared to heavy metals and pesticides due to their persistence and bioactivity.^3,4^ These compounds enter the environment through treated wastewater, landfill leachates, sewer systems, and agricultural runoff.^4–6^ Antibiotics in pharmaceutical wastewater threaten aquatic ecosystems by fostering antibiotic resistance and disrupting microbial communities.^7,8^ Conventional wastewater treatment plants often fail to remove pharmaceuticals, necessitating advanced treatment technologies to mitigate their environmental impact.^7–10^

Biochar has been extensively employed as an effective material for contaminant removal across various applications. Recent advances in biochar research highlight its significant potential for environmental remediation, with studies revealing improved contaminant removal efficiency achieved through engineered modifications and the utilization of byproducts.^11–17^ Hydrochar has recently emerged as a highly effective adsorbent for removing pharmaceutical compounds from wastewater. Its production through hydrothermal carbonization (HTC) offers several advantages, including lower energy consumption, reduced emissions compared to pyrolysis and higher char yields.^18^ In this review, hydrochar refers to the non-activated carbonaceous material produced directly from HTC. In contrast, materials subjected to chemical activation (*e.g.*, KOH, ZnCl_2_) at higher temperatures are termed hydrochar-derived activated carbon (HDAC) to reflect their transformed structure akin to activated carbons.

To further underscore the novelty and distinctiveness of this review, it is imperative to highlight its departure from existing literature, particularly in comparison to prior reviews such as Ouyang *et al.* (2020),^19^ which focused on biomass-derived activated carbons (biochars) produced *via* pyrolysis for the removal of pharmaceutical micropollutants from wastewater. Unlike the conventional high-temperature pyrolysis approach (300–700 °C)^20^ detailed in earlier works, this review pioneers an in-depth exploration of hydrochar, a carbonaceous material synthesized through hydrothermal carbonization (HTC) at significantly lower temperatures (180–250 °C).^21^ This method uniquely enables the direct processing of wet biomass feedstocks – such as sewage sludge, food waste and agricultural residues – eliminating the energy-intensive drying step required for biochar production, thereby offering a more sustainable and cost-effective alternative. The inherent differences between hydrochar and biochar stem from the thermal conversion methods and their consequent physicochemical properties. Biochar, produced *via* pyrolysis under oxygen-limited conditions, exhibits a highly aromatic and porous structure due to extensive condensation reactions. In contrast, hydrochar, formed in the presence of water under autogenous pressure, retains a higher abundance of oxygen-containing functional groups (–OH, –COOH, –C–O–) and a less ordered carbon structure.^22,23^ These characteristics result from HTC reactions such as hydrolysis, dehydration and decarboxylation, which prevent complete aromatic condensation.^24,25^

The distinctive surface chemistry and morphology of hydrochar and HDAC significantly enhance their adsorption capacity for various contaminants, particularly pharmaceuticals. Research has shown that hydrochar effectively adsorbs pharmaceuticals, positioning it as a promising material for wastewater treatment.^4,26^ Moreover, while previous studies broadly addressed pharmaceutical removal with an emphasis on adsorption kinetics and isotherms, this review narrows its focus to antibiotic pollution, a pressing global concern due to rising antimicrobial resistance. Despite these advancements, existing reviews, such as Ouyang *et al.* (2020),^27^ exhibit significant shortcomings that highlight critical research gaps. Firstly, prior studies predominantly focus on biochar derived from pyrolysis, neglecting the potential of hydrochar and its activated forms (HDAC) produced *via* HTC, which limits the exploration of sustainable solutions for wet biomass valorization – a resource abundant yet underexploited in wastewater treatment. Secondly, these reviews lack comprehensive data on the adsorption of antibiotics, a pressing concern given the global rise in antimicrobial resistance and instead emphasize broad pharmaceutical categories with limited mechanistic insights into polar contaminant removal. Thirdly, the absence of standardized comparative analyses or benchmarking against commercial adsorbents in earlier works hinders a clear understanding of hydrochar's competitive edge. Also, previous research falls short in addressing practical challenges such as regeneration efficiency, scalability and integration into hybrid treatment systems, often remaining theoretical with insufficient guidance for real-world applications. This review addresses these gaps by providing an in-depth mechanistic analysis of hydrochar and HDAC, introducing innovative applications like hybrid treatment systems and composite materials, and offering empirical data supported by standardized comparisons, thereby positioning our work as a significant advancement in the field.

Hydrochar and HDAC exhibit several advantages as an adsorbent compared to traditional materials such as activated carbon or biochars. The enhanced adsorption capability of hydrochar is mainly attributable to the rich array of surface functional groups, which facilitate mechanisms such as ion exchange and complexation for polar contaminants.^28,29^ Furthermore, the tunability of hydrochar's surface chemistry and textural properties permits the design of tailored adsorbents for specific environmental remediation applications, with research demonstrating comparable or even superior performance in aqueous media.^30^ Such attributes underscore the potential of hydrochar as a promising, eco-friendly alternative in wastewater treatment and environmental remediation, aligning with current trends toward sustainable valorization of organic waste materials.^31^

Recent advancements in 2025 have further enhanced the application of hydrochar in pharmaceutical pollution mitigation, with innovative approaches such as the use of orange peel-derived hydrochar activated with H_2_O_2_ and HCl for antibiotic removal^32^ and sewage sludge-based hydrochar optimized for ciprofloxacin adsorption.^33^ Morover, magnetic sawdust hydrochar functionalized with MOFs has shown promising results for tetracycline elimination,^34^ while steam-activated hydrochar from grape stalks has expanded its potential for pharmaceutical micropollutants.^35^ These developments, integrated into our review, highlight the evolving role of hydrochar and HDAC in addressing antibiotic resistance and complex wastewater matrices.

The review aims to comprehensively review hydrochar and HDAC production through HTC and its application as a sustainable adsorbent for removing pharmaceutical contaminants from wastewater. The review aims to assess how controlling key parameters, such as temperature and residence time, influence the physicochemical properties of hydrochar and HDAC. It further examines the effectiveness of hydrochar in adsorbing pharmaceutical compounds by analyzing its adsorption mechanisms and performance under various conditions. The article also highlights the challenges associated with hydrochar utilization, including pH sensitivity, non-selective adsorption and inefficiencies in regeneration processes. Moreover, it presents recommendations for improving hydrochar and HDAC preparation, activation and regeneration techniques to enhance its feasibility and efficiency in wastewater treatment applications. By synthesizing existing research, the review positions hydrochar as a promising solution for mitigating the environmental impact of pharmaceutical pollutants within the framework of sustainable wastewater management.

## 2. Hydrochar preparation

### 2.1. Advantages and challenges of hydrochar preparation

Hydrochar preparation through HTC offers several advantages that underscore its potential as a sustainable process. A key benefit is its ability to utilize wet biomass feedstocks, such as sewage sludge, food waste or agricultural residues, without energy-intensive drying, unlike pyrolysis-based biochar production. This compatibility reduces energy consumption and enables the valorization of high-moisture waste, contributing to circular economy goals.^31^ However, hydrochar preparation faces notable challenges that can impact its scalability and performance. The variability in feedstock composition (*e.g.*, lignin *vs.* cellulose content) leads to inconsistent hydrochar properties, complicating standardization for industrial applications.^36–38^ Additionally, while HTC is energy-efficient, subsequent activation processes (*e.g.*, KOH or ZnCl_2_ treatment) to produce HDAC and enhance porosity can be costly and require additional energy inputs, potentially offsetting sustainability benefits.^39,40^ The process also generates byproducts, such as process water with dissolved organics, which may require treatment to prevent environmental release.^41^ Moreover, achieving high recovery yields (*e.g.*, 70–99% for rice husks^42^) often comes at the expense of porosity, necessitating a trade-off between yield and performance for adsorptive applications.

### 2.2. Hydrothermal carbonization

**Fig. 1:**
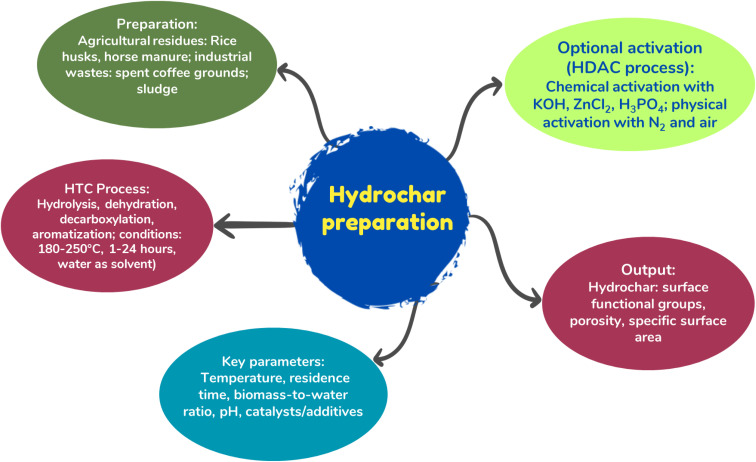
Hydrothermal carbonization process.

Fig. 1 presents a mind map illustrating the preparation of hydrochar *via* hydrothermal carbonization, summarizing key stages and influencing factors. Hydrochar is a material formed through HTC, which converts high-moisture biomass into carbonaceous solids.^43^ Hydrochar is often prepared *via* HTC using water as the solvent and reaction medium at temperatures ranging from 150 °C to 350 °C and under autogenous pressure to enrich oxygenated functional groups.^44^ The process is typically conducted at temperatures between 180 °C and 250 °C, with reaction times varying from 1 to 24 hours and pressures between 14 and 22 MPa.^45^ Two major conversion pathways are commonly utilized in HTC: (1) liquid-phase biomass conversion through mechanisms such as hydrolysis, dehydration, decarboxylation, fragmentation, polymerization and aromatization and (2) direct solid–solid conversion *via* devolatilization, intramolecular condensation, dehydration and decarboxylation.^43^

### 2.3. Main factors influencing hydrochar properties

The properties of hydrochar, a carbon-rich material produced through HTC, are significantly influenced by various factors, including feedstock type, temperature, residence time and biomass-to-water ratio. The type of feedstock is a primary factor influencing hydrochar properties. The type of feedstock significantly influences hydrochar properties, as varying proportions of cellulose, hemicellulose, and lignin in biomass sources like lignocellulosic materials and agricultural residues directly affect the hydrochar's chemical composition and characteristics. Studies have shown that the complexity of feedstocks can lead to distinct hydrochar properties, with variations in nutrient retention and adsorption capabilities depending on the inorganic content of the feedstock.^36,38,46^

Temperature is another critical parameter in the HTC process. Increased temperatures generally promote devolatilization and dehydration, which can lower hydrochar yield while altering its energy properties.^47^ Research indicates that higher temperatures can adversely affect the ion exchange capacity and the total oxygen-containing functional groups in hydrochar, which are essential for its adsorptive capabilities.^48^ Moreover, the mass yield of hydrochar is more significantly influenced by temperature than residence time, highlighting the importance of optimizing this parameter for desired hydrochar characteristics.^49,50^

The residence time during the HTC process also plays a role, albeit slightly less than temperature. Studies have shown that while longer residence times can enhance specific properties, they do not significantly affect the overall mass yield of hydrochar.^49,50^ The biomass-to-water ratio is also crucial; for example, using a higher one can lead to hydrochar with improved carbon content and porosity, desirable traits for various applications.^51^

The pH of the reaction medium significantly influences the properties of hydrochar. Acidic conditions enhance hydrolysis reactions, resulting in hydrochar with increased porosity and surface area, which are advantageous for adsorption applications.^4,52^ Conversely, alkaline conditions favor condensation reactions, producing hydrochar with different structural characteristics. Moreover, the pH affects the solubility of various components during the HTC process, further impacting the final properties of the hydrochar.

Table 1 presents the specific surface area, pore volume and recovery yield of hydrochars produced from various feedstocks, including horse manure, olive residues, sucrose, spent coffee grounds and brown algal biomass, *via* HTC conducted at temperatures ranging from 160 °C to 230 °C for 2 to 24 hours, with or without subsequent activation. Hydrochars exhibit low specific surface areas (0.0043–76 m^2^ g^−1^) and pore volumes (0.000261–0.48 cm^3^ g^−1^), yet achieve high recovery yields (up to 99% for rice husks), rendering them suitable for applications such as wastewater treatment. Chemical activation using agents such as KOH, K_2_CO_3_ or ZnCl_2_ at temperatures up to 900 °C significantly enhances specific surface areas (up to 2431 m^2^ g^−1^) and pore volumes (up to 1.14 cm^3^ g^−1^) for HDAC, albeit at the expense of reduced yields (as low as 2.3% for poplar sawdust). Hydrochars derived from sucrose and brown algal biomass demonstrate exceptional post-activation surface areas, making them well-suited for adsorption processes. Prolonged HTC durations further improve the properties of hydrochars from feedstocks such as spent coffee grounds. These results highlight the pivotal influence of feedstock selection and activation processes in optimizing hydrochar and HDAC characteristics for targeted environmental applications.

**Table 1:** Specific surface area and pore properties of hydrochar and HDAC^a^

Hydrochar/HDAC	Hydrothermal carbonization condition (tem, time)	Activated condition	Recovery yield (%)	Specific surface area (m^2^ g^−1^)	Pore volume (cm^3^ g^−1^)	References
Horse manure (HH1)	210 °C, 17 h	—	18–15	11.7–16.5	0.13–0.11	53
Horse manure (HH2)	230 °C, 17 h					
Olive residues (OH1)	210 °C, 17 h	—	45–38	3.2–1	0.04–0.08	
Olive residues (OH2)	230 °C, 17 h					
Tomato residues (TH1)	210 °C, 17 h	—	46–37	1.7–0.3	0.09–0.02	
Tomato residues (TH2)	230 °C, 17 h					
Rice husk (RH1)	210 °C, 17 h	—	99–70	49.5–49.5	0.34–0.34	
Rice husk (RH2)	230 °C, 17 h					
Raphia farinifera (FH1)	210 °C, 17 h	—	60–48	1.4–0.9	0.01–0.03	
Raphia farinifera (FH2)	230 °C, 17 h					
Human faecal simulant (EH1)	210 °C, 17 h	—	13–11	0.5–0.5	0.05–0.09	
Human faecal simulant (EH2)	230 °C, 17 h					
Sucrose	190 °C, 5 h	800 °C, 1 h (KOH activation)	—	2431	1.14	55
Sucrose	190 °C, 5 h	800 °C, 1 h (K_2_CO_3_ activation)	—	1375	0.63	
Sucrose	190 °C, 5 h	800 °C, 1 h (steam activation)	—	814	0.37	
SH600 (sucrose + KOH)	190 °C, 5 h	600 °C, 1 h	—	1169	0.57	
SH700 (sucrose + KOH)	190 °C, 5 h	700 °C, 1 h	—	1987	0.91	
SH800 (sucrose + KOH)	190 °C, 5 h	800 °C, 1 h	—	2431	1.14	
SC700 (sucrose + K_2_CO_3_)	190 °C, 5 h	700 °C, 1 h	—	987	0.44	
SC800 (sucrose + K_2_CO_3_)	(190 °C, 5h)	800 °C, 1h	—	1375	0.63	
SC900 (sucrose + K_2_CO_3_)	(190 °C, 5 h)	900 °C, 1 h	—	1200	0.59	
Rice husks	220 °C	—	—	16.92	—	54
Horse manure	220 °C	—	—	4.62	—	
Tomato waste	220 °C	—	—	0.74	—	
κ-carrageenan (HC-κ)	200 °C, 20 h	—	—	4.88	0.007	56
ι-carrageenan (HC-ι)				30.44	0.0245	
λ-carrageenan (HC-λ)				11.89	0.0107	
κ-carrageenan (HC-κ)	200 °C, 20 h	700 °C, 4 h, 1 : 4 KOH : HC	—	4.88	0.007	
ι-carrageenan (HC-ι)				30.44	0.0245	
λ-carrageenan (HC-λ)				11.89	0.0107	
Spent coffee ground	160 °C, 2 h	—	80.3	0.17	0.000261	57
	160 °C, 4 h		72.0	0.26	0.000609	
	160 °C, 6 h		67.1	0.33	0.000698	
	160 °C, 8 h		65.5	0.36	0.000716	
	160 °C, 10 h		64.7	0.93	0.004278	
	160 °C, 12 h		64.5	1.29	0.005592	
Poplar sawdust	220 °C, 8 h		—	7.5		62
		Activated at 300 °C in N_2_ (N300)	83.4	16.6	—	
		Activated at 500 °C in N_2_ (N500)	56.1	314.4		
		Activated at 700 °C in N_2_ (N700)	51.5	358.6	—	
		Activated at 300 °C in air (O300)	50.2	11.0	—	
		Activated at 500 °C in air + N (O500)	29.6	618.02	—	
		Activated at 700 °C in air + N (O700)	2.3	557.6	—	
Rice husk: hydrochloric acid-assisted 5 mol L^−1^ HCl		180 °C, 12 h	—	1.3	22	39
Brown algal	180 °C, 6 h		—	76	0.48	63
Brown algal: activation by ZnCl_2_	180 °C, 6 h	700 °C, 2 h, ZnCl_2_	—	1326	0.93	
Rice husk	180 °C, 24 h	—	—	1.80	0.012	64
Rice husk: dual-activator modification for HDAC with H_3_PO_4_ and K_2_CO_3_	180 °C, 24 h, H_3_PO_4_	800 °C, 2 h, K_2_CO_3_	—	1265.08	0.575	

a Note: “—” not specified.

#### 2.3.1. Hydrothermal carbonization of agricultural residues

The hydrochars produced from horse manure (HH1 and HH2) at temperatures of 210 °C to 230 °C for 17 hours exhibited specific surface area (SSA) ranging from 11.7 to 16.5 m^2^ g^−1^ and pore volumes between 0.11 and 0.13 cm^3^ g^−1^.^53^ The recovery yield decreased from 18% to 15% with increasing temperature, indicating a trend of thermal degradation consistent with findings in the literature, where higher temperatures often lead to increased decomposition of organic matter.^36,46^ Hydrochars derived from olive (OH1, OH2) and tomato residues (TH1, TH2) demonstrated even lower SSA, with values ranging from 3.2 to 1.0 m^2^ g^−1^ for olive residues and 1.7 to 0.3 m^2^ g^−1^ for tomato residues.^53^ The recovery yields for both feedstocks also decreased with higher temperatures, reflecting a similar trend of thermal degradation as seen in horse manure.^50^ The low SSA and pore volume of this hydrochar highlight the challenges associated with optimizing HTC conditions for feedstocks that inherently possess low carbon content. Previous studies have emphasized the importance of optimizing HTC parameters to enhance the functionality of such feedstocks, suggesting that temperature and residence time modifications could improve the yield and properties of hydrochars derived from low-carbon agricultural residues.^49,51^

In contrast, hydrochars produced from rice husks (RH1, RH2) achieved a remarkably high SSA of 49.5 m^2^ g^−1^, with recovery yields decreasing from 99% to 70% as temperature increased.^54^ The consistent pore volume of 0.34 cm^3^ g^−1^ indicates a favorable structure for adsorptive applications, supporting findings in the literature that suggest rice husk-derived hydrochars exhibit enhanced porosity and surface area, making them suitable for environmental applications such as pollutant adsorption.^38,40^ The high recovery yield, even at elevated temperatures, suggests that rice husks are a resilient feedstock that can maintain desirable properties during the HTC process, corroborating studies that highlight the effectiveness of rice husks in producing high-quality hydrochars.^40^

The hydrochars produced from raphia foraminifera and human fecal simulants (EH1, EH2; EH1, EH2) exhibited significantly lower SSA (≤1.4 m^2^ g^−1^) and pore volumes ranging from 0.01 to 0.09 cm^3^ g^−1^.^53^ The low SSA and pore volume indicate limited porosity development, which restricts their utility in high-performance material applications such as adsorbents for environmental cleanup. The recovery yields for these feedstocks reflected similar thermal degradation trends, suggesting that the inherent composition of these materials may not lend itself to the formation of porous structures during HTC. The limited adsorptive capacity of hydrochars from raphia foraminifera and human fecal simulants aligns with previous studies that have reported similar findings for low-carbon feedstocks.

The specific surface area (SSA), pore volumes and yields of hydrochars derived from various biomass residues are summarized herein, including horse manure (SSA: 11.7–16.5 m^2^ g; yield: 15–18%), olive and tomato wastes (SSA: 0.3–3.2 m^2^ g; yield: 37–46%), rice husks (SSA: 49.5 m^2^ g; yield: 70–99%) and others such as raphia and human fecal simulants (SSA: <1.4 m^2^ g^−1^). Notable discrepancies are evident, particularly the elevated SSA of rice husk hydrochars relative to the low values observed for other residues, which are attributable to differences in lignocellulosic composition: the high silica and lignin content (20–30% lignin) in rice husks confers resistance to degradation during HTC, thereby preserving structural integrity and promoting higher porosity, whereas protein-rich or low-carbon residues, such as tomato waste, undergo extensive decomposition, yielding amorphous hydrochars with reduced SSA. Contradictory findings regarding temperature effects on SSA have been reported, with specific studies indicating an increase in SSA with rising temperature (*e.g.*, for horse manure, from 11.7 m^2^ g^−1^ at 210 °C to 16.5 m^2^ g^−1^ at 230 °C, owing to enhanced devolatilization). In contrast, others document decreases attributed to pore collapse at temperatures exceeding 230 °C. Yields also exhibit inconsistencies, as rice husks maintain high values despite temperature increases, in contrast to sharp declines observed for manure and olive wastes (15–45%), arising from variations in hemicellulose content that facilitates hydrolysis in non-siliceous feedstocks. These properties directly impact application performance, with high-SSA rice husk hydrochars being well-suited for adsorption processes (*e.g.*, 51.86 mg g^−1^ for norfloxacin), whereas low-SSA tomato hydrochars exhibit limited adsorption capacity. Furthermore, measurement methodologies contribute to observed variations, as nitrogen adsorption, utilized in these investigations, may overlook macropores, potentially underestimating pore volumes in manure hydrochars (0.11–0.13 cm^3^ g^−1^) relative to mercury porosimetry data (0.2–0.6 cm^3^ g^−1^) for comparable feedstocks.

#### 2.3.2. Activation and surface modification

The surface of HDAC derived from various feedstocks, including sucrose, carrageenan, and spent coffee grounds, demonstrates the significant impact of chemical activation on enhancing the physicochemical properties of these materials. This analysis will explore the findings related to each feedstock, emphasizing the implications for potential applications in environmental remediation.

The HDAC from sucrose demonstrates remarkable SSA and pore volume improvements due to activation, particularly with KOH at elevated temperatures. The SSA increased dramatically from 814 m^2^ g^−1^ to 2431 m^2^ g^−1^, with the highest values achieved at 800 °C.^55^ This substantial enhancement in SSA indicates a significant development of porosity, which is crucial for applications requiring high adsorption capacity.^46^ The effectiveness of KOH activation in HDAC properties underscores the pivotal role of chemical modifiers in tailoring the characteristics. KOH not only facilitates the removal of volatile components but also enhances the formation of oxygen-containing functional groups, which are essential for improving the adsorptive properties of HDAC.^47,48^ Studies have shown that HDAC with higher oxygen content improves performance in removing heavy metals from aqueous solutions, highlighting the importance of activation in optimizing HDAC for specific applications.

The HDAC derived from carrageenan, subjected to HTC at 200 °C followed by KOH activation, achieved SSA up to 30.44 m^2^ g^−1^.^56^ Although these values are lower than those of sucrose-derived HDAC, the unique properties of carrageenan-based HDAC may lend themselves to niche applications, particularly in biomedicine and low-grade adsorption. The relatively modest SSA suggests that while these HDACs may not compete with higher SSA materials for traditional adsorption applications, they could be effective in specialized contexts, such as drug delivery systems or as scaffolds in tissue engineering.^51^ The lower absolute values of SSA in carrageenan-based HDAC may also reflect the inherent structural characteristics of the feedstock, which is composed of polysaccharides that may not yield the same level of porosity as more carbon-rich feedstocks like sucrose.^40^ Nonetheless, the successful activation of carrageenan hydrochars demonstrates the potential for functionalization and modification to meet specific application needs.

The HTC of spent coffee grounds resulted in hydrochars with SSA ranging from 0.17 to 1.29 m^2^ g^−1^, with recovery yields decreasing as treatment duration increased from 2 to 12 hours.^57^ This trend suggests limited porosity development under mild HTC conditions, consistent with findings in the literature indicating that spent coffee grounds typically yield hydrochars with lower surface areas than other feedstocks.^58^ The low SSA values associated with spent coffee grounds highlight the challenges of utilizing this feedstock for hydrochar production, particularly under mild HTC conditions. Previous studies have indicated that more aggressive HTC conditions or subsequent activation treatments may be necessary to enhance the porosity and surface area of hydrochars derived from coffee grounds.^59^ Moreover, bioactive compounds in spent coffee grounds, such as chlorogenic acids, present opportunities for valorization beyond adsorption applications, including potential uses in agriculture and pharmaceuticals.^60,61^

Herein, activation substantially enhances SSA of HDAC, as exemplified by sucrose activated with KOH at 800 °C (SSA: 2431 m^2^ g; pore volume: 1.14 cm^3^ g^−1^); however, discrepancies are apparent, such as the low SSA of 30.44 m^2^ g^−1^ for carrageenan under KOH activation and the range of 0.17–1.29 m^2^ g^−1^ for spent coffee grounds without activation, despite extended hydrothermal carbonization (HTC) durations (2–12 h). These variations are attributed to feedstock composition: polysaccharide-rich materials like carrageenan produce less ordered structures following HTC, thereby constraining activation efficiency, whereas simple sugars in sucrose facilitate extensive pore formation through KOH etching. Contradictory observations include KOH activation at elevated temperatures (800–900 °C) occasionally diminishing yields (*e.g.*, to 2.3% for poplar sawdust) without consistently reducing SSA, as air activation at 500 °C yields 618 m^2^ g^−1^ compared to 358 m^2^ g^−1^ at 700 °C, owing to oxidative *versus* inert atmospheric conditions. These properties influence performance, with high-SSA of HDAC demonstrating superior pharmaceutical adsorption (*e.g.*, 650 mg g^−1^ for sulfamethoxazole). In contrast, low SSA variants exhibit inferior capabilities, with discrepancies arising from activator efficacy (KOH > K_2_CO_3_ > steam). Recent investigations affirm that acidic activations (*e.g.*, HCl) augment SSA (*e.g.*, to 1322 m^2^ g^−1^ for rice husk), contrasting neutral methods by promoting ion exchange sites for polar contaminants.

#### 2.3.3. Exceptional performances

The HDAC derived from poplar sawdust exhibited remarkable increases in SSA, particularly when subjected to nitrogen (N_2_) and air activation at elevated temperatures. The SSA reached up to 618 m^2^ g^−1^ at 700 °C, indicating a significant development of porosity conducive to applications requiring high surface area.^62^ The ability to achieve high SSA through activation methods underscores the importance of optimizing processing conditions to maximize the utility of biomass-derived materials.^47^ Furthermore, the use of N_2_ and air as activation atmospheres can influence the chemical and physical properties of the resulting hydrochars, affecting their performance in specific applications.^48^

The study by Luo *et al.* (2024)^39^ provides insights into the properties of hydrochar derived from rice husks processed at 180 °C for 12 hours. The resulting hydrochar achieved SSA of 22 m^2^ g^−1^ and a pore volume of 0.08 cm^3^ g^−1^. In contrast, hydrochloric acid-assisted HDAC (5H-HC) treated with 5 mol L^−1^ HCl significantly enhanced performance metrics, achieving an SSA of 1322 m^2^ g^−1^. This dramatic improvement demonstrates the transformative effect of acid treatment in unlocking the potential of rice husk hydrochar.

The application of dual activation methods involving ZnCl_2_ or H_3_PO_4_ has resulted in HDAC with SSAs exceeding 1200 m^2^ g^−1^ and substantial pore volumes of up to 0.93 cm^3^ g^−1^.^63,64^ These results emphasize the benefits of synergistic chemical treatments, which can significantly enhance the structural properties of HDAC beyond what is achievable through single activation methods. The effectiveness of ZnCl_2_ and H_3_PO_4_ as activating agents can be attributed to their ability to facilitate the removal of volatile components and promote the formation of a porous structure during the activation process. Previous research has demonstrated that chemical activation can lead to the development of a network of micropores and mesopores, essential for enhancing the adsorptive capacity of HDAC.^38,40^ The resulting materials can be utilized in various applications, including environmental remediation, where their high surface area allows for the effective adsorption of contaminants from aqueous solutions.

This analysis highlights the significant variability in SSA and pore properties of hydrochars across different feedstocks and treatment methods. Biomass type plays a critical role, with lignocellulosic materials like rice husks outperforming protein-rich or low-carbon residues regarding SSA and porosity, making them more suitable for high-performance applications. Thermal and chemical optimization, mainly through elevated temperatures and chemical activators such as KOH and ZnCl_2_, significantly enhances the structural properties of HDAC, underscoring their importance in creating high-performance adsorbents. Moreover, while hydrochar derived from refined materials like sucrose achieves exceptionally high SSA, agricultural and industrial waste feedstocks offer a more sustainable alternative by contributing to waste valorization and circular economy objectives.

Analyzing hydrochar produced from various feedstocks under different HTC conditions reveals significant insights into its elemental composition and potential applications. The data summarized in Table 2 indicates that the C, H, N, O and S contents, along with atomic ratios such as H/C, O/C, and N/C, are critical in defining the properties of hydrochar and HDAC. These properties are essential for evaluating the suitability of hydrochar for various applications, including environmental remediation. This review highlights elevated SSAs in HDAC, as exemplified by poplar sawdust under air activation (618 m^2^ g^−1^), rice husk with HCl activation (1322 m^2^ g^−1^), and dual-activated variants (1265 m^2^ g^−1^), in contrast to discrepancies with raw hydrochars (*e.g.*, poplar at 7.5 m^2^ g^−1^). These enhancements are explained by synergistic activation processes (*e.g.*, ZnCl_2_/H_3_PO_4_), which facilitate volatile removal and mesopore formation. However, atmospheric conditions introduce variations – air oxidation yields higher SSA at 500 °C but promotes collapse and lower SSA at 700 °C compared to inert N_2_ environments. Contradictory findings include brown algal biomass with ZnCl_2_ activation achieving 1326 m^2^ g^−1^, comparable to rice husk dual activation despite differing feedstocks, attributable to algal polysaccharides *versus* husk silica content; additionally, certain studies report yield trade-offs (*e.g.*, 2.3% for high-SSA materials). Performance implications encompass enhanced adsorption capacities (*e.g.*, 208 mg g^−1^ for tetracycline in magnetic HDAC), yet discrepancies arising from activation methods (single *versus* dual) influence reusability, with efficiencies exceeding 85% in optimized cases contrasted against below 50% in others.

**Table 2:** Summary of hydrochar and HDAC properties^a^

Feedstock	Hydrothermal carbonization conditions	Activation	Carbon content (% wt)	Specific surface area (m^2^ g^−1^)	Pore volume (cm^3^ g^−1^)	Key property notes	References
Horse manure	210–230 °C, 17 h	—	40–50	11.7–16.5	0.11–0.13	Moderate carbon (C) content, low surface area, high yield	53
Olive residues	210–230 °C, 17 h	—	45–55	1.0–3.2	0.04–0.08	Stable C, limited porosity for basic applications	53
Sucrose	190 °C, 5 h	KOH, 800 °C	70–80	2431	1.14	High C content, exceptional surface area post-activation	55
Spent coffee grounds	160 °C, 2–12 h	—	50–60	0.17–1.29	0.000261–0.005592	Increase C and porosity over a longer time	57
Brown algal biomass	180 °C, 6–24 h	ZnCl_2_, 700 °C	60–70	76–1326	0.48–0.93	High C enhances activation potential	63
Poplar sawdust	220 °C, 8 h	Air/N_2_, 500–700 °C	55–65	11.0–618.02	—	C Content supports moderate porosity post-activation	62
Orange peels	200–250 °C, 4–8 h	H_2_O_2_/HCl	80–90	100–500	0.3–0.4	High C content, moderate surface area post-activation	32
Microalgae biomass	180–220 °C, 6–12 h	—	65–75	50–200	0.2–0.35	High C enhances activation potential	65

a Note: “—” not specified.

#### 2.3.4. Elemental composition trends

The carbon content of hydrochars is a pivotal factor governing their physicochemical properties and applicability across the environmental domain, as demonstrated by studies on various feedstocks in Table 2. Higher carbon content, typically ranging from 60–80% wt, as observed in sucrose and brown algal biomass, significantly enhances the structural robustness and porosity development during HTC and subsequent activation processes.^55,63^ For instance, sucrose hydrochar with an estimated 70–80% wt carbon, when activated with KOH at 800 °C to produce HDAC, achieves an exceptional specific surface area of 2431 m^2^ g^−1^ and a pore volume of 1.14 cm^3^ g^−1^.^55^ This high carbon content facilitates the formation of a stable carbon framework, which supports extensive microporous and mesoporous structures during activation, making such HDAC ideal for applications like pollutant adsorption.^55^ Similarly, brown algal biomass, with a carbon content of 60–70% wt, exhibits a surface area of 1326 m^2^ g^−1^ and a pore volume of 0.93 cm^3^ g^−1^ after ZnCl_2_ activation at 700 °C, underscoring the role of carbon in enhancing activation potential.^63^ In contrast, hydrochars with lower carbon content, such as horse manure (40–50% wt) and olive residues (45–55% wt), yield modest surface areas of 11.7–16.5 m^2^ g^−1^ and 1.0–3.2 m^2^ g^−1^, respectively and pore volumes of 0.11–0.13 cm^3^ g^−1^ and 0.04–0.08 cm^3^ g^−1^, limiting their use to less demanding applications.^53^ The moderate carbon content of spent coffee grounds (50–60% wt) results in low surface areas (0.17–1.29 m^2^ g^−1^) and negligible pore volumes (0.000261–0.005592 cm^3^ g^−1^) without activation, but longer HTC durations improve these properties slightly, suggesting a partial influence of carbon stabilization.^57^ Poplar sawdust, with 55–65% wt carbon, achieves a surface area of up to 618.02 m^2^ g^−1^ after activation at 500–700 °C. This indicates that even intermediate carbon levels can support significant porosity with appropriate activation.^62^ Hydrochar derived from microalgae biomass, processed at 180–220 °C for 6–12 hours, possesses a carbon content of 65–75% wt, a specific surface area of 50–200 m^2^ g^−1^, and a pore volume of 0.2–0.35 cm^3^ g^−1^, demonstrating significant potential for improved activation and adsorption efficiency.^65^ In contrast, H_2_O_2_/HCl-activated orange peel HDAC, synthesized at 200–250 °C for 4–8 hours, features a high carbon content of 80–90% wt, with a surface area ranging from 100–500 m^2^ g^−1^ and a pore volume of 0.3–0.4 cm^3^ g^−1^, indicating its suitability for enhanced activation and effective adsorption applications.^32^ Higher carbon content also enhances thermal stability during activation, reducing structural collapse at elevated temperatures (500–800 °C), which is critical for maintaining high yields of porous HDAC.^55,62,63^

Carbon content in hydrochars ranges from 40 to 90 wt%, as exemplified by horse manure (40–50%) and orange peels (80–90%), with elevated concentrations in refined feedstocks promoting enhanced SSA post-activation (*e.g.*, sucrose at 70–80% enabling 2431 m^2^ g^−1^). Discrepancies are evident, including the porosity limitations in low-carbon manure (11.7 m^2^ g^−1^) relative to high-carbon algae (60–70%, yielding 1326 m^2^ g^−1^), stemming from initial compositional factors—proteins facilitate carbon volatilization, whereas polysaccharides support its retention. Contradictory findings reveal that activation typically increases carbon content (*e.g.*, from 55–65% in poplar to elevated levels following N_2_ activation), although specific investigations report declines attributable to oxidation. These attributes influence adsorption efficacy, with higher carbon content correlating to greater hydrophobicity, thereby favoring the sequestration of non-polar pharmaceuticals.

#### 2.3.5. Atomic ratios

The atomic ratios of hydrochar, particularly the H/C, O/C, and N/C ratios, are crucial for understanding its chemical properties and potential applications. These ratios provide insights into the structural features and functional properties of hydrochar, which can be tailored for specific uses in environmental and energy applications.

The H/C ratio is a key indicator of the degree of aromaticity and hydrophobicity in hydrochar. Lower H/C ratios (*e.g.*, 0.15–0.16 for spent coffee grounds) indicate a higher degree of aromaticity and a reduced presence of hydrogen functional groups.^63,66,67^ Conversely, hydrochar derived from feedstocks like brown algae or rice husks typically exhibits higher H/C ratios, reflecting incomplete carbonization and a more significant proportion of aliphatic structures. The literature supports that higher H/C ratios correlate with lower thermal stability.^68^ The O/C ratio is a critical indicator of the degree of oxidation in hydrochar, which influences its thermal stability and functional applications. A lower O/C ratio signifies a more advanced carbonization process characterized by reduced oxygen-containing functional groups. For instance, hydrochar produced from horse manure (HH1) at 210 °C for 17 hours exhibits an oxygen content of 24.7%, resulting in a moderate O/C ratio. When the temperature is increased to 230 °C for the same duration (HH2), the oxygen content decreases to 19.7%, reflecting a lower O/C ratio due to intensified dehydration and decarboxylation reactions. This observation is consistent with findings in the scientific literature, which indicate that elevated temperatures reduce oxygen content, thereby improving the calorific value of hydrochar and enhancing its suitability for solid fuel applications.^53^

In a similar vein, HDAC derived from κ-carrageenan (HC-κ) and ι-carrageenan (HC-ι) activated at 700 °C demonstrates oxygen contents of 24.813% and 23.951%, respectively. Conversely, feedstocks such as rice husks, which possess an oxygen content of 37.69%, exhibit a higher O/C ratio. The N/C ratio is a critical parameter for evaluating nitrogen enrichment in hydrochar, which has significant implications for its potential applications in environmental remediation. Elevated N/C ratios indicate improved nitrogen retention during the hydrothermal carbonization process, often linked to the utilization of protein-rich feedstocks or specific chemical modifications. For example, hydrochar derived from horse manure exhibits nitrogen contents of 0.8% (HH1) and 0.3% (HH2), resulting in a moderate N/C ratio that suggests its viability as a slow-release nitrogen fertilizer.^53^ Furthermore, under comparable conditions, hydrochar produced from olive waste and κ-carrageenan demonstrates slightly higher nitrogen contents of 1.58% and 0.356%, respectively.^56^ Conversely, hydrochars with negligible nitrogen content, such as those derived from rice husks (*N* = not detectable) or spent coffee grounds (*N* = approximately 0.02–0.61%, depending on processing duration), exhibit lower N/C ratios. This characteristic renders them less suitable for direct agricultural applications; however, they may still hold potential for carbon sequestration or adsorption-based applications.^57^

Research advancements in 2025 elucidate the influence of feedstock composition and elemental ratios on the adsorption efficiency of hydrochar for antibiotic removal. H_2_O_2_/HCl-activated orange peel HDAC, synthesized at 200–250 °C for 20 hours, exhibits a composition of 65.3% C and 29.0% O, with elemental ratios of H/C (0.955), O/C (0.333), and N/C (0.006). The elevated oxygenated functional groups enhance its adsorption capacity, achieving 1.971 mg g^−1^ for sulfamethoxazole.^32^ Steam-activated grape stalk hydrochar, prepared at 200–260 °C for 20 hours, contains 72.1% C and 22.4% O, with H/C (0.799) and O/C (0.233) ratios. This composition facilitates a high adsorption capacity of 25.19 mg g^−1^ for diclofenac, primarily through pore-filling mechanisms.^35^ Citric acid-modified sewage HDAC, synthesized at 180–240 °C for 20 hours, comprises 58.9% C and 33.8% O, with H/C (1.242) and O/C (0.43) ratios, enabling an adsorption capacity of 17.76 mg g^−1^ for ciprofloxacin *via* inner-sphere complexation.^33^ Metal–organic framework (MOF)-functionalized magnetic pine sawdust hydrochar, processed at 200–250 °C for 20 hours, contains 68.5% C and 26.1% O, with H/C (0.788) and O/C (0.286) ratios. This material achieves an exceptional adsorption capacity of 169.23 mg g^−1^ for tetracycline, driven by chemisorption and π–π interactions, with over 90% reusability.^34^ Also, magnetic sewage sludge HDAC, synthesized at 180–250 °C for 4–8 hours with Fe_3_O_4_ incorporation, exhibits 55.0% C and 35.0% O, with H/C (1.2) and O/C (0.477) ratios, indicating significant potential for pollutant remediation.^69^ The hydrogen-to-carbon (H/C) ratios (0.15–1.5) and oxygen-to-carbon (O/C) ratios (0.2–1.6) exhibit considerable variation, with diminished values signifying enhanced aromaticity (*e.g.*, spent coffee grounds at 0.15–0.16 H/C); however, discrepancies arise, such as elevated H/C ratios in sewage sludge (1.24), attributable to incomplete carbonization processes. Contradictory observations indicate that acidic conditions reduce O/C ratios through decarboxylation mechanisms, whereas alkaline environments promote condensation reactions, resulting in pH-dependent compositional trends. Nitrogen-to-carbon (N/C) ratios are elevated in protein-rich feedstocks (*e.g.*, horse manure at 0.002–0.61), which augments their suitability for fertilizer applications but contrasts with the preference for low-nitrogen materials in adsorption contexts.

This analysis situates the findings regarding hydrochar's chemical properties within the broader scientific context, emphasizing the implications of atomic ratios such as H/C, O/C and N/C for various applications. The low H/C and O/C ratios observed in hydrochar indicate advanced carbonization processes, which align with findings from previous studies that highlight hydrochar's suitability for adsorptive applications. Specifically, the reduction in these ratios suggests increased aromaticity and hydrophobicity, making hydrochar more favorable for use as adsorbents in environmental remediation efforts.^70,71^ The literature supports this assertion, noting that hydrochars with lower H/C and O/C ratios exhibit enhanced thermal stability and energy density, critical for their application as solid fuels.^72,73^ In contrast, high N/C ratios are associated with improved nitrogen retention during hydrothermal carbonization, particularly in hydrochars derived from nitrogen-rich feedstocks. By correlating these atomic ratios with their respective functional and structural implications, this analysis reinforces the versatility of hydrochar across multiple domains. Hydrochar's potential extends beyond energy applications to include environmental remediation, highlighting its role as a sustainable material in addressing contemporary challenges such as waste management.^74,75^ Furthermore, the ability to tailor hydrochar properties through adjustments in feedstock and processing conditions underscores its adaptability for specific applications, ranging from carbon sequestration to developing advanced materials for various industrial uses.^76^

#### 2.3.6. The percentage of C, H, N, O and S in hydrochar and HDAC

The elemental composition of hydrochar, encompassing carbon (C), hydrogen (H), nitrogen (N), oxygen (O), and sulfur (S), is critical for understanding its chemical properties, potential applications and suitability for adsorption materials. The following discussion integrates data from Table 3 with relevant literature to elucidate these aspects. Carbon is the predominant element in hydrochar, significantly influencing its energy content and thermal stability. For instance, hydrochar derived from horse manure processed at 210 °C for 17 hours (HH1) exhibits a carbon content of 70.4%, which increases to 77.7% when processed at 230 °C for the same duration (HH2). This trend underscores the enhanced carbonization at elevated temperatures, where the removal of volatile matter results in a carbon-rich structure. Such high carbon content is consistent with findings by Charlson (2017),^53^ who reported similar enhancements in the calorific value of hydrochar with increasing processing temperatures. In contrast, HDAC produced from κ-carrageenan (HC-κ) activated at 700 °C displays a slightly lower carbon percentage of 70.734%. This reduction can be attributed to KOH's chemical activation process, which introduces additional functional groups that may displace some carbon content. Furthermore, hydrochar derived from olive waste, processed at 220 °C, retains a notably high carbon content of 83.71%. This observation indicates that specific feedstocks with lower inherent moisture or volatile content can achieve higher carbon concentrations, even under moderate processing conditions.

**Table 3:** Elemental composition and atomic ratios of hydrochar and HDAC production^a^

Hydrochar/HDAC feedstock	Hydrothermal condition	Activation	Automatic ratios			C (%)	H (%)	N (%)	O (%)	S (%)	References
Hydrocharthermal carbonization			H/C	O/C	N/C						
Horse manure (HH1)	210 °C, 17 h	—	—	—	—	70.4	—	0.8	24.7	—	53
Horse manure (HH2)	230 °C, 17 h	—	—	—	—	77.7	—	0.3	19.7	—	
Rice husks		220 °C	—	—	—	54.3	—	—	37.69	—	54
Horse manure		220 °C	—	—	—	74.52	—	1.52	23.57	—	
Tomato waste		220 °C	—	—	—	87.84	—	0.64	11.51	—	
Olive waste		220 °C	—	—	—	83.71	—	1.58	14.71	—	
κ-carrageenan (HC-κ)	200 °C, 20 h	700 °C, 4 h, 1 : 4 KOH : HC	—	—	—	70.734	4.453	—	24.813	—	56
ι-carrageenan (HC-ι)	200 °C, 20 h	700 °C, 4 h, 1 : 4 KOH : HC	—	—	—	71.214	4.479	0.356	23.951	—	
λ-carrageenan (HC-λ)	200 °C, 20 h	700 °C, 4 h, 1 : 4 KOH : HC	—	—	—	67.141	4.112	—	28.747	—	
Spent coffee grounds (HC-2h)	160 °C, 2 h	—	0.16	0.58	0.61	0.16	0.58	0.16	0.58	—	57
Spent coffee grounds (HC-4h)	160 °C, 4 h	—	0.16	0.55	0.59	0.16	0.55	0.16	0.55	—	
Spent coffee grounds (HC-6h)	160 °C, 6 h	—	0.16	0.51	0.54	0.16	0.51	0.16	0.51	—	
Spent coffee grounds (HC-8h)	160 °C, 8 h	—	0.15	0.50	0.53	0.15	0.50	0.15	0.50	—	
Spent coffee grounds (HC-10h)	160 °C, 10 h	—	0.16	0.48	0.52	0.16	0.48	0.16	0.48	—	
Spent coffee grounds (HC-12h)	160 °C, 12h	—	0.15	0.48	0.51	0.15	0.48	0.15	0.48	—	
Loquat cores (LC)	160 °C, 2 h	—	0.16	—	0.021	41.21	6.65	0.87	50.40	0.87	67
HDAC (HC-Cit 3M)	200 °C, axid citric 3M	—	0.075	—	0.002	63.97	4.84	0.12	31.07	<0.30	
Brown algal (SW)		—	1.5	1.6	—	35	5	56	3	1	63
Hydrothermal carbonization from brown algal	180 °C, 6 h	—	1.1	0.8	—	57	6	35	2	1	
Activation by ZnCl_2_ (HTC-ZnCl_2_)	180 °C, 6 h	700 °C, 2 h, ZnCl_2_	0.25	0.2	—	75	2	17	2	1	
Orange peels HDAC	200–250 °C, 20 h	H2O2/HCl	0.95	0.33	0.333	65.3	5.2	0.5	29.0	—	32
Grape stalks hydorochar	200–260 °C, 20 h	Steam	0.79	0.23	0.008	72.1	4.8	0.7	22.4	—	35
Sewage sludge HDAC	180–240 °C, 20 h	Citric acid	1.24	0.43	0.017	58.9	6.1	1.2	33.8	0.2	33
Pine sawdust magnetic HDAC	200–250 °C, 20 h	MOF (ZIF-8)	0.78	0.28	0.011	68.5	4.5	0.9	26.1	—	34
Sewage sludge magnetic HDAC	180–250 °C, 4–8 h	Magnetic (Fe_3_O_4_)	1.2	0.47	0.015	55.0	5.5	1.0	35.0	0.3	69

a Note: “—” not specified.

The hydrogen content in hydrochar significantly influences its H/C ratio, an essential indicator of the material's aromaticity and hydrophobicity. For example, hydrochar derived from rice husks exhibits a hydrogen content that is not detectable, suggesting that an advanced dehydration process occurs during HTC. This finding implies that the HTC conditions effectively remove hydrogen, leading to a more carbon-rich and hydrophobic structure. In contrast, HDAC produced from κ-carrageenan (HC-κ) displays a higher hydrogen content of 4.453%. This increase can be attributed to the introduction of hydrogen-functional groups during the chemical activation process with KOH. The presence of these functional groups enhances the material's hydrophilicity, which is consistent with the findings reported by Nogueira *et al.* (2018),^56^ who emphasized the role of chemical activation in modifying the functional properties of HDAC.

The oxygen content in hydrochar plays a critical role in determining its O/C ratio, which in turn influences the material's energy density and hydrophilicity. Hydrochar derived from rice husks exhibits a notably high oxygen content of 37.69%. This elevated level suggests that the carbonization process is incomplete, resulting in a higher concentration of oxygen-containing functional groups, such as hydroxyl and carboxyl groups. These functional groups enhance the hydrophilicity of the hydrochar, making it more suitable for applications. However, hydrochar produced from horse manure demonstrates significantly lower oxygen levels, with values of 24.7% for hydrochar processed at 210 °C (HH1) and 19.7% at 230 °C (HH2). These lower oxygen contents reflect a more advanced carbonization process facilitated by the higher processing temperatures. The reduction in oxygen content correlates with an increase in the material's energy density, making it more suitable for use as an absorbent. Furthermore, HDAC derived from κ-carrageenan (HC-κ), activated at 700 °C, retains an oxygen content of 24.813%. This finding indicates that, despite the chemical activation process, there is a partial retention of oxygen-functional groups. This observation is consistent with the findings reported by Nogueira *et al.* (2018),^56^ which emphasize the influence of chemical activation on the functional properties of HDAC.

The sulfur content in hydrochar is influenced by the feedstock type and the specific processing conditions employed during HTC. Although most hydrochar samples presented in Table 3 do not explicitly report sulfur content, it is generally understood that sulfur is retained in small quantities throughout the HTC process. For instance, HDAC derived from κ-carrageenan (HC-κ), processed at a temperature of 700 °C, exhibits negligible sulfur retention. This observation suggests that effective desulfurization occurs at elevated temperatures during the chemical activation process, significantly reducing sulfur content. Minimizing sulfur levels is particularly advantageous, as high sulfur content in hydrochar and HDAC can lead to undesirable emissions when the material is used as a fuel. The implications of sulfur retention and desulfurization are critical for evaluating the environmental impact of hydrochar when utilized in energy applications. Lower sulfur content enhances the suitability of hydrochar for combustion processes, reducing the potential for sulfur dioxide emissions, which are associated with acid rain and other environmental concerns.

Elemental compositions exhibit C contents ranging from 55 to 87% (*e.g.*, horse manure: 70–77%; rice husks: 54%), H from undetectable levels to 6.65%, O from 11 to 50%, with generally low S and N concentrations in most instances. Discrepancies are apparent, including elevated C in olive waste (83%) relative to rice husks (54%), stemming from carbon-rich precursor materials; contradictory trends encompass oxygen reduction with increasing temperature (from 24.7% to 19.7% in manure), contrasted by retention in activated variants (*e.g.*, 24% in carrageenan). These attributes influence adsorption performance: higher O content promotes hydrogen bonding interactions for pharmaceutical sequestration, whereas lower O levels enhance hydrophobicity.

## 3. Hydrochar and HDAC application for pharmaceutical removal from wastewater

### 3.1. Target contaminants and removal performance

**Fig. 2:**
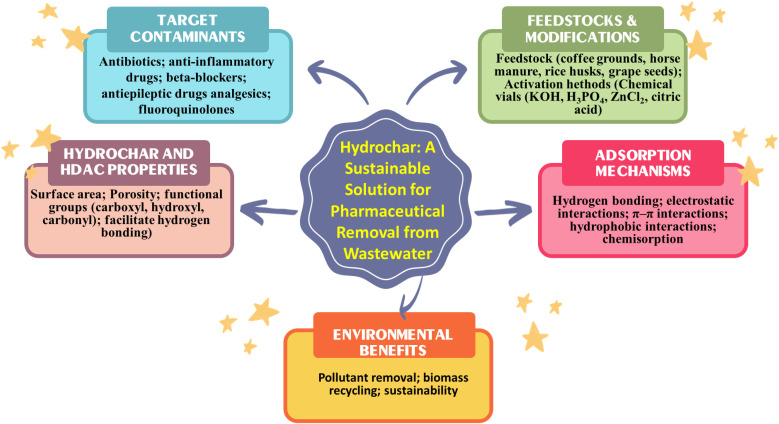
Hydrochar: a sustainable solution for pharmaceutical removal from wastewater.

Hydrochar, synthesized through the HTC of various biomass feedstocks, represents a promising and sustainable method for adsorbing pharmaceutical contaminants from wastewater. The efficacy of hydrochar in removing these contaminants is not uniform; instead, it exhibits significant variability depending on the specific type of pharmaceutical compound targeted. Notable categories of these contaminants include antibiotics, anti-inflammatory drugs, beta-blockers, antiepileptic medications and analgesics. The observed differences in removal performance can be attributed to several factors related to the physicochemical properties of hydrochar and HDAC. Key characteristics such as surface area, porosity and the presence of functional groups play a critical role in determining the adsorption capacity of hydrochar and HDAC. Also, the nature of the interactions between hydrochar and the pollutants – ranging from van der Waals forces to hydrogen bonding and electrostatic interactions – further influences the overall removal efficiency. In the following sections, a detailed examination of these target contaminants will be conducted and their removal efficiencies will be analyzed in the context of hydrochar and HDAC's properties. This analysis will be supported by empirical data presented in Fig. 2 and Table 4, which illustrates the comparative effectiveness of hydrochar and HDAC in adsorbing various pharmaceutical contaminants.

**Table 4:** Summary of pharmacy removal by hydorchar and HDAC^a^

Hydrochar/HDAC preparation	Specific properties	Adsorbate	Max. Sorption capacity (mg g^−1^)	Isotherm adsorption experimental conditions	Mechanisms	References
**1. Antibiotics**						
Horse manure HDAC: covered with ultrapure water, carbonized at 220 °C for 2 h, filtered, dried, demineralized with 0.1 M HCl, rinsed, filtered and dried	BET surface area: 4.62 m^2^ g; oxygen and nitrogen-containing functional groups	Ciprofloxacin	0.0018	Contact time: 1–3 min; hydrochar mass: 50 ± 2.5 mg; pollutant conc.: 10 μg L; volume: 10 mL	Hydrophobic interactions, hydrogen bonding	39
		Sulfamethoxazole	0.0011	Contact time: 1–3 min; hydrochar mass: 50 ± 2.5 mg; pollutant conc.: 10 μg L; volume: 10 mL	Hydrophobic interactions, hydrogen bonding	39
Tomato waste HDAC: covered with ultrapure water, carbonized at 220 °C for 2 h, filtered, dried, demineralized with 0.1 M HCl, rinsed, filtered and dried	BET surface area: 0.74 m^2^ g^−1^	Ciprofloxacin	0.0015	Contact time: 1–3 min; hydrochar mass: 50 ± 2.5 mg; pollutant conc.: 10 μg L; volume: 10 mL	Hydrophobic interactions, hydrogen bonding	39
		Sulfamethoxazole	0.0008	Contact time: 1–3 min; hydrochar mass: 50 ± 2.5 mg; pollutant conc.: 10 μg L; volume: 10 mL	Hydrophobic interactions, hydrogen bonding	39
Olive waste HDAC: Covered with ultrapure water, carbonized at 220 °C for 2 h, filtered, dried, demineralized with 0.1 M HCl, rinsed, filtered, dried	BET surface area: 0.65 m^2^ g^−1^	Ciprofloxacin	0.0006	Contact time: 1–3 min; hydrochar mass: 50 ± 2.5 mg; pollutant conc.: 10 μg L; volume: 10 mL	Hydrophobic interactions, hydrogen bonding	39
		Sulfamethoxazole	0.0006	Contact time: 1–3 min; hydrochar mass: 50 ± 2.5 mg; pollutant conc.: 10 μg L; volume: 10 mL	Hydrophobic interactions, hydrogen bonding	39
Spent coffee ground hydorchar: hydrothermal carbonization at 160 °C for 10 h	Surface area: 0.17 m^2^ g; pore volume: 0.000261 cm^3^ g; abundant oxygenated groups	Sulfadiazine	0.2951	Contact time: 24 h; adsorbent dosage: 100 mg; pollutant conc.: 5–2000 μg L; volume: 30 mL	Hydrogen bonding, monolayer/multilayer adsorption (Langmuir/Freundlich)	54
		Sulfamethoxazole	0.7406	Contact time: 24 h; adsorbent dosage: 100 mg; pollutant conc.: 5–2000 μg L; volume: 30 mL	Hydrogen bonding, monolayer/multilayer adsorption (Langmuir/Freundlich)	54
		Sulfadiazine	0.1215	Contact time: 120 min; adsorbent dosage: 3.3 g L; pollutant conc.: 500 μg L^−1^	—	66
		Sulfamethoxazole	0.1301	Contact time: 120 min; adsorbent dosage: 3.3 g L; pollutant conc.: 500 μg L^−1^	—	66
Grape seed HDAC: KOH activation at 750 °C		Sulfamethoxazole	650.8	Contact time: 100 min; adsorbent dosage: 0.3 g L; pollutant conc.: 25 mg L^−1^		
Grape seed HDAC: FeCl_3_ activation at 750 °C		Sulfamethoxazole	147.4	—		78
Grape seed HDAC: H_3_PO_4_ activation at 350 °C		Sulfamethoxazole	128.6	—		78
Orange peels HDAC: HTC + H_2_O_2_/HCl activation	BET surface area: 79.5 m^2^ g; mesopore area: 74.6 m^2^ g; enhanced –OH, carboxyl groups	Sulfamethoxazole (SMX)	1.971	Contact time: 24 h; pH: 6–7; pollutant conc.: 40 μM; dosage: 0.2 g L^−1^	Chemisorption, pore retention	32
Rice husk HDAC: hydrothermal treatment	Surface area: 1.3 m^2^ g; low pore volume	Levofloxacin	61.0	pH: neutral; contact time: 12 h; pollutant conc.: 5–200 mg L; dosage: 50 mg/100 mL	Electrostatic interaction, hydrogen bonding	89
Rice husk 5H-HC: one-step hydrothermal with HCl	Surface area: 22 m^2^ g; increased C=O content	Levofloxacin	107.0	pH: neutral; contact time: 12 h; pollutant conc.: 5–200 mg L; dosage: 50 mg/100 mL	Electrostatic interaction, hydrogen bonding, π–π stacking	89
Poplar sawdust with phosphate: hydrothermal at 240 °C	Large surface area; well-developed pores; abundant oxygenated groups	Ciprofloxacin	98.38	pH: 6; contact time: 24 h; pollutant conc.: 200–500 mg L; hydrochar mass: 0.2 g; volume: 200 mL	Physical adsorption, electrostatic interaction, hydrogen bonding, π–π interactions	89
Sewage sludge HDAC: HTC (180–240 °C) + citric acid modification	BET surface area: ∼5.68 m^2^ g; oxygenated groups	Ciprofloxacin	17.76	Contact time: 24 h; pH: 5–9; pollutant conc.: 10–50 mg L; dosage: 0.5–1 g L	Inner-sphere complexation	33
Rice husk: hydrothermal at 200 °C	—	Norfloxacin	9.68	Pollutant conc.: 80 mg L; adsorbent dosage: 2 g L^−1^	—	62
Rice husk: hydrothermal at 200 °C + acidification	—	Norfloxacin	10.362	Pollutant conc.: 80 mg L; adsorbent dosage: 2 g L^−1^	—	62
Rice husk HDAC: hydrothermal at 200 °C + H_2_O_2_	—	Norfloxacin	51.86	Pollutant conc.: 80 mg L; adsorbent dosage: 2 g L^−1^	—	62
Corn stalk: hydrothermal at 200 °C + acidification	—	Norfloxacin	22.232	Pollutant conc.: 80 mg L; adsorbent dosage: 2 g L^−1^	—	62
Corn stalk HDAC: hydrothermal at 200 °C + H_2_O_2_	—	Norfloxacin	13.756	Pollutant conc.: 80 mg L; adsorbent dosage: 2 g L^−1^	—	62
Corn stalk: hydrothermal at 200 °C + high temperature aging	—	Norfloxacin	10.853	Pollutant conc.: 80 mg L; adsorbent dosage: 2 g L^−1^	—	62

**2. Beta-blockers**						
Various hydrochars (horse manure, olive residues, tomato residues, rice husk, raphia farinifera, human fecal simulant): hydrothermal at 210–230 °C	—	Atenolol	78–99% (not in mg g^−1^)	Pollutant conc.: 100 ng L^−1^	—	53

**3. Anti-inflammatory**						
Horse manure HDAC: covered with ultrapure water, carbonized at 220 °C for 2 h, filtered, dried, demineralized with 0.1 M HCl, rinsed, filtered, dried	BET surface area: 4.62 m^2^ g; oxygen and nitrogen-containing functional groups	Diclofenac	0.0018	Contact time: 1–3 min; hydrochar mass: 50 ± 2.5 mg; pollutant conc.: 10 μg L; volume: 10 mL	Hydrophobic interactions, hydrogen bonding	39
Tomato waste HDAC: covered with ultrapure water, carbonized at 220 °C for 2 h, filtered, dried, demineralized with 0.1 M HCl, rinsed, filtered, dried	BET surface area: 0.74 m^2^ g^−1^	Diclofenac	0.0017	Contact time: 1–3 min; hydrochar mass: 50 ± 2.5 mg; pollutant conc.: 10 μg L; volume: 10 mL	Hydrophobic interactions, hydrogen bonding	39
Olive waste HDAC: covered with ultrapure water, carbonized at 220 °C for 2 h, filtered, dried, demineralized with 0.1 M HCl, rinsed, filtered, dried	BET surface area: 0.65 m^2^ g^−1^	Diclofenac	0.0014	Contact time: 1–3 min; hydrochar mass: 50 ± 2.5 mg; pollutant conc.: 10 μg L; volume: 10 mL	Hydrophobic interactions, hydrogen bonding	39
HDAC (HC-Cit 3M): acid-assisted hydrothermal with citric acid	Increased carbon content; abundant oxygenated groups; improved porosity	Diclofenac	76%	pH: 6.1–6.4; contact time: 7 h; pollutant conc.: 30 mg L; dosage: 2.5 g L^−1^	Physical adsorption, π–π interactions, hydrogen bonding	63
Loquat cores: untreated, natural state	Very low surface area; minimal functional groups	Diclofenac	20%	pH: 6.1–6.4; contact time: 90 min; pollutant conc.: 15–120 mg L; dosage: 10 g L^−1^	Limited physical adsorption	63
Grape stalks HDAC HTC + steam activation	BET surface area: 500–1000 m^2^ g; multi-layered porous structure	Diclofenac	25.19	Contact time: 24 h; pH: 5–7; pollutant conc.: 10–50 mg L; dosage: 0.2–1 g L	Pore-filling, H-bonding	35

**4. Antiepileptic/anticonvulsants**						
Various hydrochars (horse manure, olive residues, tomato residues, rice husk, raphia farinifera, human fecal simulant): hydrothermal at 210–230 °C	—	Carbamazepine	71–99% (not in mg g^−1^)	Pollutant conc.: 100 ng L^−1^	—	53
Poplar HDAC: H_3_PO_4_ hydrothermal + K_2_CO_3_ pyrolysis activation	Surface area: 1265.08 m^2^ g; aromatic porous structure; pore volume: 0.575 cm^3^ g^−1^	Carbamazepine	376.11	Pollutant conc.: 30–70 mg L; dosage: 100 mg L; contact time: 24 h	—	62

**5. Analgesics**						
Sucrose HDAC: 1.5 M, 190 °C for 5 h; KOH activation at 800 °C	Surface area: 2431 m^2^ g; abundant oxygenated groups	Paracetamol	513.5	Contact time: 24 h; hydrochar mass: 6 mg; pollutant conc.: 45–300 mg L; volume: 9–30 mL	Micropore network, surface chemistry	67
		Iopamidol	1049.6	Contact time: 24 h; hydrochar mass: 6 mg; pollutant conc.: 45–300 mg L; volume: 9–30 mL	Micropore network, surface chemistry	67
Sucrose HDAC: 1.5 M, 190 °C for h; K_2_CO_3_ activation at 800 °C	Surface area: 1375 m^2^ g; oxygen/nitrogen functional groups	Paracetamol	471.8	Contact time: 24 h; hydrochar mass: 6 mg; pollutant conc.: 45–300 mg L; volume: 9–30 mL	Not specified	67
		Iopamidol	150.9	Contact time: 24 h; hydrochar mass: 6 mg; pollutant conc.: 45–300 mg L; volume: 9–30 mL	—	67
Sucrose HDAC: 1.5 M, 190 °C for 5 h; steam activation at 800 °C	Surface area: 814 m^2^ g; graphitic nitrogen species	Paracetamol	267.7	Contact time: 24 h; hydrochar mass: 6 mg; pollutant conc.: 45–300 mg L; volume: 9–30 mL	—	67
		Iopamidol	472.4	Contact time: 24 h; hydrochar mass: 6 mg; pollutant conc.: 45–300 mg L; volume: 9–30 mL	—	67
Horse manure HDAC: covered with ultrapure water, carbonized at 220 °C for 2 h, filtered, dried, demineralized with 0.1 M HCl, rinsed, filtered, dried	BET surface area: 4.62 m^2^ g; oxygen and nitrogen-containing functional groups	Paracetamol	0.0003	Contact time: 1–3 min; hydrochar mass: 50 ± 2.5 mg; pollutant conc.: 10 μg L; volume: 10 mL	Hydrophobic interactions, hydrogen bonding	39
Tomato waste HDAC: covered with ultrapure water, carbonized at 220 °C for 2 h, filtered, dried, demineralized with 0.1 M HCl, rinsed, filtered, dried	BET surface area: 0.74 m^2^ g^−1^	Paracetamol	0.0002	Contact time: 1–3 min; hydrochar mass: 50 ± 2.5 mg; pollutant conc.: 10 μg L; volume: 10 mL	Hydrophobic interactions, hydrogen bonding	39
Olive waste HDAC r: covered with ultrapure water, carbonized at 220 °C for 2 h, filtered, dried, demineralized with 0.1 M HCl, rinsed, filtered, dried	BET surface area: 0.65 m^2^ g^−1^	Paracetamol	0.0001	Contact time: 1–3 min; hydrochar mass: 50 ± 2.5 mg; pollutant conc.: 10 μg L; volume: 10 mL	Hydrophobic interactions, hydrogen bonding	39
HDAC (HC-Cit 3M): acid-assisted hydrothermal with citric acid	Increased carbon content; abundant oxygenated groups; improved porosity	Antipyrine	76%	pH: 6.1–6.4; contact time: 7 h; pollutant conc.: 30 mg L; dosage: 2.5 g L^−1^	Physical adsorption, π–π interactions, hydrogen bonding	63
Loquat cores: untreated, natural state	Very low surface area; minimal functional groups	Antipyrine	5.59%	pH: 6.1–6.4; contact time: 90 min; pollutant conc.: 15–120 mg L; dosage: 10 g L^−1^	Limited physical adsorption	63
Pine sawdust magnetic HDAC: HTC + MOF (ZIF-8) functionalization	BET surface area: 200–800 m^2^ g; ZIF-8 functionalized groups	Tetracycline	169.23	Contact time: 24 h; pH: 7; pollutant conc.: 10–50 mg L; dosage: 0.5 g L^−1^	Chemisorption, π–π interactions	65

a Note: “—” Not specified.

#### 3.1.1. Antibiotics

Antibiotics are recognized as some of the most persistent and environmentally hazardous pharmaceutical pollutants, primarily due to their extensive usage and potential to contribute to antimicrobial resistance. Recent studies have highlighted the promising adsorption capabilities of hydrochar and HDAC for various antibiotics, including sulfamethoxazole (SMX), ciprofloxacin and sulfadiazine (SDZ). For instance, hydrochar derived from spent coffee grounds, processed at 160 °C for 10 hours, demonstrated an impressive adsorption capacity of 740.6 μg g^−1^ for SMX and 295.1 μg g^−1^ for SDZ. These results were obtained under conditions involving a contact time of 24 hours and pollutant concentrations ranging from 5 to 2000 μg L^−1^.^54^ The high adsorption efficiency observed in this case can be attributed to functional groups such as carboxyl and hydroxyl on the hydrochar surface, which facilitate the formation of strong hydrogen bonds with the antibiotic molecules.^77^ Similarly, HDAC activated with KOH from grape seeds, processed at a significantly higher temperature of 750 °C, achieved an even greater adsorption capacity of 650.8 mg g^−1^ for SMX. This enhancement in performance is mainly due to the increased porosity and surface area of the activated hydrochar compared to its unactivated counterparts.^78^ Such findings underscore the importance of hydrochar modification techniques to produce HDAC, which can significantly improve its adsorption properties through structural enhancements. However, it is noteworthy that simpler hydrochars, such as those derived from horse manure and tomato waste, exhibited markedly lower adsorption capacities for ciprofloxacin, measuring only 1.8 μg g^−1^ and 1.5 μg g^−1^, respectively. These lower capacities are primarily attributed to their limited surface areas, with BET surface area measurements of 4.62 m^2^ g^−1^ and 0.74 m^2^ g^−1^, respectively. The adsorption mechanisms in these cases appear to rely predominantly on hydrophobic interactions and hydrogen bonding, which are less effective than the interactions facilitated by the functional groups in more complex hydrochar.^39^

Significant differences in adsorption capacities across studies – ranging from 1.5 μg g^−1^ (ciprofloxacin, horse manure) to 650.8 mg g^−1^ (SMX) – stem from variations in feedstock composition and activation methods. Spent coffee grounds, rich in lignin, facilitate enhanced functional groups (–OH, –COOH) and superior SSA, thereby promoting hydrogen bonding for SMX/SDZ (740.6 μg g^−1^ and 295.1 μg g^−1^, respectively), whereas horse manure/tomato waste (SSA 0.74–4.62 m^2^ g^−1^) is susceptible to pore collapse due to high protein/low-carbon content, yielding low capacities (1.5–1.8 μg g^−1^) for ciprofloxacin. KOH activation (at 750 °C) of grape seeds elevates SSA (>1000 m^2^ g^−1^) and enhances π–π interactions, thereby improving chemisorption for SMX. Contradictions in adsorption mechanisms – hydrogen bonding in non-activated hydrochar *versus* π–π stacking in HDAC – reflect dependencies on SSA and aromaticity. Ciprofloxacin necessitates synergistic interactions (electrostatic/π–π), which are effective solely in activated forms.

The findings from these studies align with the broader scientific literature, which emphasizes the critical role of hydrochar properties – such as surface area, porosity and functional group composition – in determining its effectiveness as an adsorbent for pharmaceutical contaminants. For instance, research has shown that modifying hydrochar through various activation methods to produce HDAC can significantly improve adsorption capacities for various pollutants.^37^ Furthermore, the environmental implications of utilizing hydrochar for wastewater treatment are substantial, as it not only aids in the removal of hazardous substances but also promotes the recycling of biomass waste, contributing to a more sustainable approach to waste management.^79^

#### 3.1.2. Anti-inflammatory drugs

Anti-inflammatory drugs, including diclofenac and ibuprofen, are frequently detected in wastewater due to their widespread use and persistence in the environment. Removing these pharmaceuticals is critical, given their potential adverse effects on aquatic ecosystems and human health. Recent studies have demonstrated that hydrochar derived from horse manure exhibits modest removal capabilities for diclofenac, achieving an adsorption capacity of approximately 1.8 μg g^−1^ under a contact time of 1–3 minutes and a pollutant concentration of 10 μg L^−1^.^39^ Despite the relatively low BET surface area of this hydrochar, the presence of oxygen- and nitrogen-containing functional groups facilitates adsorption primarily through hydrogen bonding, consistent with findings in the literature that highlight the importance of surface chemistry in adsorption processes.^77^ Moreover, chemically enhanced hydrochars to make HDAC have shown significantly improved adsorption performance. For example, HC-Cit 3M, produced *via* acid-assisted hydrothermal treatment with citric acid, achieved a remarkable diclofenac removal efficiency of 76%. This enhancement is attributed to increased porosity and a higher density of oxygenated functional groups, which are known to enhance the adsorption capacity of HDAC for various contaminants.^63^ Such modifications underscore the critical role of surface treatment in optimizing HDAC to remove pharmaceutical pollutants.

Significant variations in diclofenac adsorption performance – from a low capacity of 1.8 μg g^−1^ (horse manure hydrochar) to a removal efficiency of 76% (HC-Cit 3M) – reflect inconsistencies arising from feedstock properties and treatment methods. Horse manure, characterized by high protein and low carbon content, produces hydrochar with a low SSA of 4.62 m^2^ g^−1^, limiting physical adsorption and relying on weak hydrogen bonding, resulting in poor capacity. In contrast, HC-Cit 3M (citric acid-assisted HDAC) enhances porosity and oxygenated functional groups (–COOH), improving hydrogen bonding and ion exchange, thereby achieving a 76% removal efficiency. Contradictions in adsorption mechanisms – hydrogen bonding in both cases, but supplemented by physical adsorption *via* mesopores in HC-Cit 3M – stem from citric acid promoting dehydration, which increases SSA compared to conventional hydrothermal carbonization. Diclofenac, an anionic compound (p*K*_a_ ∼4.2), exhibits weak interactions with non-activated hydrochar at pH >6 due to electrostatic repulsion, whereas HC-Cit 3M mitigates pH dependency. These discrepancies underscore the need to select thermally stable feedstocks and optimize mild activation methods (*e.g.*, citric acid) to balance adsorption capacity and efficiency.

The findings from these studies align with the broader scientific literature that emphasizes the importance of HDAC properties in determining its effectiveness as an adsorbent. For instance, introducing functional groups through chemical activation has been shown to significantly enhance the adsorption capacities of HDAC for various pollutants, including pharmaceuticals.^80^ Moreover, the relationship between hydrochar and HDAC surface area and adsorption capacity has been well-documented, with increased surface area often correlating with improved removal efficiencies.^37^ Moreover, the kinetic studies indicate that the adsorption process is time-dependent, with longer contact times generally leading to higher adsorption capacities, as seen in other studies focusing on diclofenac removal.^79^ This suggests that optimizing contact time and pollutant concentration could further enhance the efficacy of hydrochar and HDAC in wastewater treatment applications.

#### 3.1.3. Beta-blockers

Beta-blockers, such as atenolol and propranolol, are increasingly recognized as significant pharmaceutical contaminants in wastewater due to their widespread use and persistence in the environment. Recent studies have demonstrated that hydrochar and HDAC derived from various feedstocks – including rice husks, horse manure and human fecal simulants – exhibit removal efficiencies for atenolol ranging from 81% to 99%, depending on the specific feedstock and processing conditions employed.^53^ The high removal efficiency observed can be primarily attributed to the electrostatic interactions between the positively charged beta-blockers and the negatively charged functional groups on the hydrochar surface. This mechanism is consistent with findings in the literature that highlight the importance of surface charge and functional group composition in the adsorption of cationic contaminants.^77^ The effectiveness of hydrochar and HDAC in removing beta-blockers is further supported by the diverse feedstocks utilized in their production. For instance, hydrochars derived from agricultural residues often possess unique surface characteristics that enhance their adsorption capabilities. Various functional groups, including carboxyl and hydroxyl, contribute to adsorption by facilitating electrostatic interactions and hydrogen bonding.^81^ This aligns with previous research indicating that the physicochemical properties of hydrochar and HDAC, such as surface area and porosity, play a crucial role in their ability to adsorb pharmaceutical contaminants.^80^

Moreover, the processing conditions during hydrochar production significantly influence its adsorption performance. Studies have shown that optimizing parameters such as temperature, pressure and treatment time can enhance the porosity and surface area of hydrochar, thereby increasing its capacity to adsorb contaminants.^37^ For example, hydrochars produced under higher temperatures exhibit improved structural properties, which can lead to enhanced adsorption efficiencies for a range of pollutants, including beta-blockers.^79^ The findings regarding the adsorption of beta-blockers by hydrochar underscore the potential of this material as an effective adsorbent in wastewater treatment applications. The ability to tailor hydrochar properties through selecting feedstock and optimizing processing conditions presents a viable strategy for enhancing the removal of pharmaceutical contaminants from wastewater. As the scientific community continues to explore the mechanisms underlying hydrochar and HDAC adsorptions, it is essential to consider the implications of these findings for developing sustainable wastewater treatment technologies.

#### 3.1.4. Antiepileptic drugs

Antiepileptic drugs (AEDs), particularly carbamazepine, present significant challenges in wastewater treatment due to their chemical stability and low reactivity, which complicates their removal from the environment. This stability is a concern as these compounds can persist in aquatic systems, potentially leading to adverse ecological effects and human health risks. Recent studies have highlighted the potential of HDAC as an effective adsorbent for these pollutants. HDAC activated with H_3_PO_4_ and K_2_CO_3_ has demonstrated remarkable adsorption capacities, with a reported surface area of 1265 m^2^ g^−1^ and an adsorption capacity of 376.11 mg g^−1^ for carbamazepine.^62^ This high efficiency can be attributed to the presence of π–π interactions between the aromatic structures of carbamazepine and the graphitic surfaces of the HDAC, alongside a hydrogen bonding mechanism.

Significant variations in carbamazepine adsorption performance—capacity of 376.11 mg g^−1^ on hydrochar-derived activated carbon (HDAC) (H_3_PO_4_ + K_2_CO_3_, SSA 1265 m^2^ g^−1^) compared to non-activated hydrochar (*e.g.*, 1.8 μg g^−1^ for ciprofloxacin, SSA 4.62 m^2^ g^−1^) – stem from activation methods and feedstock properties. Dual activation (H_3_PO_4_ + K_2_CO_3_) generates graphitic surfaces and mesopores, enhancing π–π interactions and physical adsorption, which are well-suited to the aromatic structure of carbamazepine. In contrast, non-activated hydrochar (*e.g.*, from horse manure) relies on hydrogen bonding but is limited by low SSA, resulting in poor capacity. Lignin-rich feedstocks, such as poplar sawdust, maintain porous structures post-activation, unlike protein-rich feedstocks prone to pore collapse. Differences in SSA, π–π interactions and feedstock composition account for the superior performance of HDAC.

In the context of the broader scientific literature, the effectiveness of HDAC aligns with findings from studies on carbon nanotubes and other carbonaceous materials, which have also shown high adsorption capacities for various organic pollutants.^82^ The ability of these materials to adsorb pharmaceuticals is not only a function of their surface area but also their chemical properties, such as the presence of functional groups that can enhance interaction with target molecules.^83^ The implications of these findings are significant for the development of advanced materials for wastewater treatment, suggesting that optimizing the chemical activation process could lead to even greater efficiencies in the removal of persistent contaminants like carbamazepine from aquatic environments.

#### 3.1.5. Analgesics

The adsorption of pharmaceuticals, particularly analgesics like paracetamol, onto hydrochars has garnered significant attention due to the potential of hydrochars to mitigate water pollution. Hydrochars are carbon-rich materials produced through HTC and their adsorption capacities can vary dramatically based on their source and preparation methods. For instance, hydrochars derived from organic waste, such as horse manure and olive waste, exhibit limited adsorption capacities for paracetamol, recorded at 0.3 μg g^−1^ and 0.1 μg g^−1^, respectively.^39^ This low capacity is likely attributed to their inherently low surface areas and pore volumes, which restrict the availability of active sites for adsorption. In contrast, hydrochars that undergo chemical activation, such as KOH-activated sucrose hydrochar, demonstrate significantly enhanced adsorption capacities. The KOH-activated sucrose HDAC achieves an impressive adsorption capacity of 513.5 mg g^−1^ for paracetamol, starkly contrasting with the basic hydrochars mentioned earlier. This enhancement can be attributed to the increased surface area, measured at 2431 m^2^ g^−1^ and a microporous network that provides more active sites for interaction with paracetamol molecules.^67^ The presence of abundant oxygenated functional groups on the surface of HDAC facilitates hydrogen bonding and electrostatic interactions, which are crucial for effective adsorption.^84^

The structural characteristics of hydrochar and HDAC play a pivotal role in their adsorption capabilities. For example, KOH treatment increases the surface area and modifies the surface morphology, creating a rough texture with numerous small voids. This roughness enhances the effective contact area between the HDAC and adsorbate, facilitating adsorption.^26,85^ Furthermore, the functional groups introduced during the activation process can interact favorably with the target molecules, as seen in studies where HDAC modified with cationic minerals exhibited improved adsorption properties for various contaminants.^81^ Moreover, the adsorption mechanisms are complex and involve multiple interactions, including hydrophobic interactions, hydrogen bonding and electrostatic attractions. Hydrochar with higher carbon content and hydrophobic surfaces has been shown to effectively adsorb aromatic pollutants, indicating that surface chemistry is a critical factor in determining adsorption performance.^86,87^ The interplay between surface area, functional group density and the nature of the adsorbate must be carefully considered when designing hydrochar and HDAC for specific adsorption applications.

#### 3.1.6. Fluoroquinolones

Hydrochars with high porosity and graphitic content efficiently remove fluoroquinolones, including ciprofloxacin and levofloxacin. Rice husk-derived hydrochar removed 61 mg g^−1^ of levofloxacin *via* hydrogen bonding and electrostatic interactions. In contrast, its acid-treated counterpart, HCl-*co*-treated HDAC (5H-HC), demonstrated an improved capacity of 107 mg g^−1^, supported by its enhanced surface area (22 m^2^ g^−1^) and increased carbonyl functional group density.^88^ A particularly noteworthy performance was observed with ZnCl_2_-activated HDAC, which achieved a 416.7 mg g^−1^ adsorption capacity for ciprofloxacin. This HDAC, with a surface area of 1326 m^2^ g^−1^, utilized a combination of π–π interactions, chemisorption and hydrogen bonding, showcasing the potential of advanced activation methods to enhance removal efficiency significantly.^64^

Removing fluoroquinolones, such as ciprofloxacin and levofloxacin, from aqueous solutions using hydrochar and HDAC has emerged as a promising approach in wastewater treatment. The efficiency of hydrochars in adsorbing these pharmaceuticals is closely linked to their structural properties, particularly porosity and functional group density. For instance, rice husk-derived hydrochar demonstrated a 61 mg g^−1^ adsorption capacity for levofloxacin, primarily through hydrogen bonding and electrostatic interactions.^89^ However, when this hydrochar underwent acid treatment (HCl-*co*-treated HDAC, designated as 5H-HC), its adsorption capacity increased significantly to 107 mg g^−1^.^89^ This enhancement is attributed to the increased surface area and a higher density of carbonyl functional groups, which facilitate stronger interactions with the fluoroquinolone molecules.^90^ The performance of HDAC can be further amplified through advanced activation methods. A notable example is the ZnCl_2_-activated HDAC, which achieved an exceptional adsorption capacity for ciprofloxacin. This HDAC, with a surface area of 1326 m^2^ g^−1^, utilized a combination of π–π interactions, chemisorption, and hydrogen bonding, showcasing the potential of advanced activation methods to enhance removal efficiency significantly.^64^ Such a combination of interactions underscores the possibility of chemical activation to enhance the removal efficiency of HDAC for pharmaceutical contaminants significantly.^84^

Significant variations in fluoroquinolone adsorption performance – from 61 mg g^−1^ (rice husk hydrochar) to 107 mg g^−1^ HCl-*co*-treated HDAC and 416.7 mg g^−1^ (ZnCl_2_-activated HDAC) – arise from differences in activation methods and feedstock properties. Rice husk hydrochar, characterized by low SSA, relies on hydrogen bonding and electrostatic interactions, resulting in limited capacity. HCl treatment increases SSA to 22 m^2^ g^−1^ and enhances carbonyl functional groups, improving hydrogen bonding and yielding a capacity of 107 mg g^−1^. ZnCl_2_-activated HDAC, with an SSA of 1326 m^2^ g^−1^, develops graphitic surfaces that promote π–π interactions and chemisorption, optimizing performance for ciprofloxacin at 416.7 mg g^−1^. Lignin-rich rice husk facilitates a porous structure post-activation, in contrast to protein-rich feedstocks prone to pore collapse. Variations in SSA, functional groups, and adsorption mechanisms (hydrogen bonding *versus* π–π interactions) account for the superior performance of HDAC.

The underlying adsorption mechanisms are critical for understanding how hydrochar and HDAC can be optimized for specific contaminants. The presence of functional groups, such as carbonyls and hydroxyls, plays a vital role in facilitating adsorption through various interactions. For example, the increased density of oxygen-containing functional groups on the hydrochar and HDAC surfaces enhances their capacity to adsorb positively charged contaminants, including fluoroquinolones.^85^ The structural characteristics imparted by activation processes can lead to a more heterogeneous surface, which may improve the accessibility of adsorption sites.^91^ Moreover, the adsorption behavior of hydrochar and HDAC can be influenced by the initial concentration of the target contaminants. At lower concentrations, adsorption tends to occur primarily on the outer surfaces of the hydrochar and HDAC, which may result in lower overall capacities due to limited penetration into the porous structure.^81^ Conversely, at higher concentrations, the driving force for mass transfer increases, allowing for deeper penetration and higher adsorption capacities. This phenomenon highlights the importance of optimizing hydrochar and HDAC properties and the operational conditions to maximize adsorption efficiency.^87^

Recent studies in 2025 provide detailed insights into the adsorption performance of hydrochar and HDAC for pharmaceutical contaminant removal. HDAC derived from H_2_O_2_/HCl-activated orange peel, with a BET surface area of 79.5 m^2^ g^−1^, demonstrates a maximum adsorption capacity of 1.971 mg g^−1^ for sulfamethoxazole. This process is primarily driven by chemisorption and pore retention mechanisms, with optimal performance observed under pH 6–7 conditions.^32^ Similarly, steam-activated grape stalk HDAC, possessing a significantly higher BET surface area of 500–1000 m^2^ g^−1^, achieves an enhanced adsorption capacity of 25.19 mg g^−1^ for diclofenac. The removal mechanism is attributed to pore-filling and hydrogen bonding, effective within a pH range of 5–7.^35^ In contrast, citric acid-modified sewage sludge hydrochar, with a lower BET surface area of approximately 5.68 m^2^ g^−1^, exhibits a notable adsorption capacity of 17.76 mg g^−1^ for ciprofloxacin. This performance is facilitated by inner-sphere complexation and remains effective across a broader pH range of 5–9.^33^ Furthermore, metal–organic framework (MOF)-functionalized magnetic pine sawdust hydrochar, with a BET surface area ranging from 200–800 m^2^ g^−1^, achieves an exceptional adsorption capacity of 169.23 mg g^−1^ for tetracycline. This is driven by chemisorption and π–π interactions, with the material demonstrating over 90% reusability at pH 7.^34^ These findings highlight the critical role of surface chemistry and structural properties in optimizing hydrochar-based adsorbents for pharmaceutical remediation.

### 3.2. Factors affecting adsorption efficiency

#### 3.2.1. Feedstock and preparation methods

**Fig. 3:**
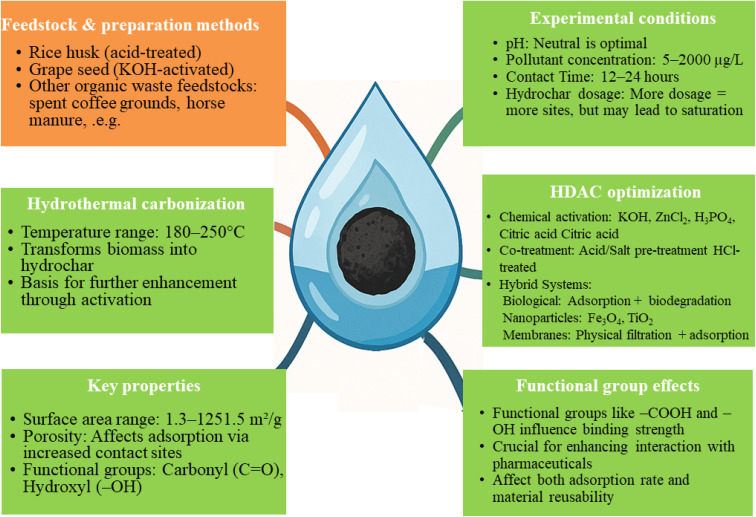
Factors enhancing hydrochar and HDAC's adsorption efficiency for pharmaceuticals.

The adsorption efficiency of hydrochar and HDAC for pharmaceutical contaminants is significantly influenced by the choice of feedstock and the preparation methods employed. These factors critically determine the surface area, pore structure and functional group composition of the final hydrochar and HDAC products, which are essential for optimizing its performance in contaminant removal (Fig. 3). Recent studies have highlighted that tailoring these characteristics is crucial for enhancing the hydrochar's ability to target specific contaminants effectively.^92,93^

The feedstock utilized in hydrochar production plays a pivotal role in defining its adsorption properties. For instance, agricultural residues such as rice husks and grape seeds yield hydrochars with moderate surface areas and functional group densities. Research indicates that rice husk hydrochar processed at 200 °C exhibited a surface area of 1.3 m^2^ g^−1^ and a limited adsorption capacity of 9.68 mg g^−1^ for norfloxacin. However, subsequent acid treatment significantly enhanced its performance; acid-treated rice husk HDAC achieved a surface area of 22 m^2^ g^−1^ and an adsorption capacity of 51.86 mg g^−1^, attributed to increased carbonyl group density.^62,89^ Similarly, grape seed HDAC activated with KOH at 750 °C demonstrated an exceptional adsorption capacity of 650.8 mg g^−1^ for sulfamethoxazole, linked to its highly porous structure and functionalized surface.^78^ Furthermore, organic waste feedstocks, such as spent coffee grounds and horse manure, have effectively produced hydrochars rich in oxygen- and nitrogen-containing functional groups. These groups facilitate hydrogen bonding and electrostatic interactions with pharmaceutical contaminants. For example, spent coffee ground HDAC processed at 160 °C for 10 hours achieved significant adsorption capacities of 740.6 μg g^−1^ for sulfamethoxazole and 295.1 μg g^−1^ for sulfadiazine, underscoring the importance of feedstock chemical composition.^54^

The preparation methods employed also significantly influence the characteristics of hydrochar and HDAC. The HTC is particularly advantageous for wet feedstocks, as it operates under mild reaction conditions, resulting in higher hydrochar yields than pyrolysis, which produces biochar.^94^ The process parameters, including temperature and residence time, are critical; for instance, increasing the temperature can enhance the surface area and porosity of the hydrochar, improving its adsorption capabilities.^95^ Moreover, modifications such as chemical activation with agents like KOH can further improve the surface area and functional group density of HDAC, thereby increasing the adsorption capacity for various.^93,96^

The processing conditions, including temperature, duration and activation methods, are critical determinants of HDAC properties, influencing its effectiveness in adsorbing pharmaceutical contaminants. Higher carbonization temperatures typically enhance the aromaticity of hydrochar, reduce its oxygen content, and increase its hydrophobicity, characteristics that are particularly advantageous for the adsorption of hydrophobic pharmaceutical compounds. For instance, rice husk hydrochar produced at 200 °C exhibited limited adsorption performance for norfloxacin. Still, its capacity was significantly improved following oxidative modification with H_2_O_2_, which likely increased the density of functional groups conducive to adsorption.^62^ This aligns with findings that suggest modifications can enhance the surface characteristics of HDAC, thereby improving its adsorption capabilities.^97^ Moreover, the duration of processing is equally important, as longer carbonization times facilitate the complete decomposition of volatile components, resulting in thermally stable materials with enhanced adsorption properties. For example, the extended carbonization of spent coffee ground HDAC has improved its structural integrity and adsorption efficiency.^54^ The relationship between processing duration and adsorption capacity is further corroborated by studies demonstrating that extended treatment facilitates the development of a more porous structure enhancing the capture of contaminants from aqueous solutions.^98^

Activation methods also play a significant role in HDAC properties. Chemical activation, particularly with agents such as KOH or H_2_O_2_, has been shown to significantly increase the surface area and porosity of HDAC, thereby enhancing its adsorption capacity for various contaminants.^62^ For instance, KOH activation of grape seed HDAC resulted in a marked increase in its adsorption capacity for sulfamethoxazole, demonstrating the effectiveness of chemical activation in optimizing HDAC for specific applications. Moreover, introducing functional groups through activation can enhance the interaction between HDAC and pharmaceutical contaminants, further improving adsorption performance.^99^

Enhancing HDAC's performance through chemical activation is a well-documented phenomenon in the literature, where various activating agents significantly improve the surface area and introduce specific functional groups that facilitate adsorption processes. For instance, HDAC activated with ZnCl_2_ at 750 °C has demonstrated a remarkable surface area of 1326 m^2^ g^−1^, correlating with an impressive adsorption capacity of 416.7 mg g^−1^ for ciprofloxacin. This enhanced performance is primarily attributed to chemisorption and π–π interactions, as Zhong *et al.* (2025)^64^ highlighted. The role of ZnCl_2_ as an effective activating agent is further supported by findings that indicate its ability to catalyze the degradation of cellulose components and promote the formation of larger pores on HDAC surfaces, thereby increasing its porosity and surface area.^100^ Similarly, KOH activation has been shown to produce highly porous structures in HDAC derived from grape seeds, which significantly enhances its capacity to adsorb antibiotics such as sulfamethoxazole. The effectiveness of KOH in creating a well-developed porous network has been corroborated by studies indicating that chemically activated carbons exhibit substantial mesopore and micropore content, which is crucial for the adsorption of emerging pollutants.^101^ The specific surface area of KOH-activated HDAC can reach values as high as 1251.5 m^2^ g^−1^, demonstrating the potential of this activation method to improve adsorption characteristics.^90^ Moreover, acid treatments, particularly citric acid, have been shown to enhance HDAC's adsorption efficiency by introducing oxygenated functional groups. For instance, citric acid-treated HDAC (HC-Cit 3M) achieved a 76% removal efficiency for diclofenac, which can be attributed to improved hydrogen bonding and electrostatic interactions from these functional groups.^63^ This aligns with the broader understanding that introducing functional groups through chemical activation increases surface area and enhances the interaction between the adsorbent and the target contaminants.^41^

#### 3.2.2. Experimental conditions

The adsorption efficiency of hydrochar and HDAC for pharmaceutical contaminants is significantly influenced by various experimental conditions, including pH, pollutant concentration, contact time, hydrochar dosage and solution volume (Fig. 3). These parameters dictate the interaction mechanisms between hydrochar or HDAC and pollutants, ultimately affecting the overall removal performance. Understanding these interactions is crucial for optimizing hydrochar and HDAC applications in wastewater treatment.

The pH of the solution is particularly critical in determining adsorption efficiency, as it influences both the surface charge of hydrochar and the ionization state of pharmaceutical contaminants. For instance, the adsorption of levofloxacin onto rice husk hydrochar was found to be optimal at neutral pH, where the zwitterionic form of levofloxacin facilitated strong electrostatic interactions and hydrogen bonding with the hydrochar's functional groups, achieving an adsorption capacity of 107 mg g^−1^.^89^ Similarly, HDAC modified with KOH exhibited a remarkable adsorption capacity for ciprofloxacin at pH 7, attributed to a combination of electrostatic interactions and π–π stacking.^89^ Deviations from this optimal pH can reduce adsorption efficiency, as contaminants may undergo deprotonation or protonation, which diminishes their affinity for the HDAC surface.^18^

The adsorption performance of hydrochar and HDAC is significantly influenced by both pollutant concentration and contact time, as evidenced by various studies in the scientific literature. At elevated pollutant concentrations, the gradient driving force for adsorption increases, which enhances the utilization of available adsorption sites on the hydrochar and HDAC. However, it is crucial to note that excessively high concentrations can lead to site saturation, thereby diminishing the overall removal efficiency of the adsorbent. For instance, Weidemann *et al.* (2018)^54^ demonstrated that spent coffee ground HDAC achieved adsorption capacities of 740.6 μg g^−1^ for sulfamethoxazole and 295.1 μg g^−1^ for sulfadiazine within a concentration range of 5–2000 μg L^−1^, with a contact time of 24 hours necessary to reach equilibrium. Similarly, rice husk HDAC treated with HCl showed effective adsorption of levofloxacin with pollutant concentrations ranging from 5 to 200 mg L^−1^, achieving optimal performance within 12 hours.^89^ The relationship between contact time and adsorption capacity is also well-documented. As contact time increases, adsorption capacity typically rises until it reaches a plateau, indicating equilibrium. For example, Oumabady *et al.* found that the adsorption of diclofenac by sludge-derived hydrochar increased significantly over 15 hours, after which a decline in adsorption capacity was observed, suggesting that equilibrium had been achieved.^89^ This phenomenon is consistent across various hydrochar types and pollutants, as seen in the work of Yin *et al.* (2021),^87^ who utilized rice husk hydrochars and noted that a contact time of 720 min was adequate for achieving a stable equilibrium state in the removal of multiple contaminants, including organic compounds.

Analyzing hydrochar or HDAC dosage and solution volume regarding adsorption efficiency is crucial for optimizing the removal of contaminants from aqueous solutions. Hydrochar or HDAC dosage significantly influences the availability of adsorption sites; higher dosages generally increase the number of active sites, which enhances the removal efficiency for low-concentration pollutants. For instance, the adsorption capacity of KOH-treated HDAC has been reported to reach high values for various contaminants, underscoring the necessity of optimizing HDAC quantity to balance efficiency and cost-effectiveness.^78,90,102^ However, it is essential to note that excessively high HDAC dosages can lead to overlapping active sites, which may reduce the pollutant uptake per unit of hydrochar, thereby diminishing the overall adsorption efficiency.^81,91^ This phenomenon is corroborated by findings that indicate a saturation point in adsorption capacity, where additional hydrochar does not proportionally increase contaminant removal.^103^ Moreover, the solution volume is pivotal in adsorption by affecting the diffusion and interaction dynamics between contaminants and hydrochar. A study utilizing ZnCl_2_-treated HDAC demonstrated that a solution volume of 40 mL allowed for sufficient interaction time, achieving a 416.7 mg g^−1^ capacity for ciprofloxacin under neutral pH conditions.^64^ This suggests that an optimal solution volume is necessary to facilitate effective contact between the HDAC and contaminants, thereby maximizing adsorption efficiency. The interaction time is also critical; as adsorption time increases, the capacity rises until equilibrium is reached, indicating that both h HDAC dosage and solution volume must be carefully managed to achieve optimal results.^37,104^

#### 3.2.3. Optimization approaches

The optimization of hydrochar for the removal of pharmaceutical contaminants is a multifaceted challenge that necessitates the enhancement of its physicochemical properties and the integration of hybrid treatment systems (Fig. 3). Recent studies underscore two primary strategies: chemical activation and co-treatment with acids or salts, alongside incorporating complementary processes, which have significantly boosted HDAC's adsorption efficiency.

Chemical activation is a prevalent method employed to augment the surface area, porosity and functional group density of HDAC. Activating agents such as KOH, ZnCl_2_ and H_3_PO_4_ are instrumental in creating micro- and mesoporous structures that enhance adsorption capacity. Co-treatment with acids or salts refines HDAC's adsorption properties by modifying its surface charge and introducing additional functional groups. For example, citric acid-treated HDAC (HC-Cit 3M) achieved a 76% removal efficiency for diclofenac, a result attributed to the oxygenated functional groups that promote hydrogen bonding and electrostatic interactions.^63^ Acid-treated rice husk HDAC (5H-HC) also exhibited improved performance, adsorbing 107 mg g^−1^ of levofloxacin, which was linked to an increased density of carbonyl groups and a surface area of 22 m^2^ g^−1^.^89^ These modifications enhance the adsorption capacity and improve the interaction dynamics between HDAC and pharmaceutical contaminants, as evidenced by the increased removal efficiencies observed in various studies.^105,106^

In addition to enhancing hydrochar and HDAC properties, integrating it into hybrid treatment systems can overcome limitations such as site saturation and incomplete contaminant removal. Hybrid systems combining adsorption with biological processes allow hydrochar or HDAC to serve as both an adsorbent and a substrate for microbial communities, enabling simultaneous adsorption and biodegradation of pharmaceuticals. HDAC functionalized with nanoparticles, such as Fe_3_O_4_ or TiO_2_, acts as an adsorbent and a catalyst, enabling dual mechanisms for pollutant removal. For instance, ZnCl_2_-treated HDAC, with abundant lactonic groups and high surface area, is particularly effective in synergy with oxidative processes for removing fluoroquinolones like ciprofloxacin.^64^ Additionally, incorporating hydrochar into filtration membranes provides a dual benefit of physical separation and chemical adsorption, making it highly effective for eliminating micropollutants from complex wastewater matrices. HDAC that is functionalized with nanoparticles, such as Fe_3_O_4_ or TiO_2_, serves as an adsorbent, enabling dual mechanisms for pollutant removal.^87,107^ This highlights the potential of HDAC to be tailored for specific contaminants through appropriate modifications.

Moreover, integrating hydrochar into filtration membranes offers a dual benefit of physical separation and chemical adsorption, making it particularly effective for removing micropollutants from complex wastewater matrices. The physical properties of hydrochar, such as its porosity and surface area, play a critical role in its adsorption capacity. Studies have shown that hydrochars derived from various feedstocks, including agricultural residues, exhibit different adsorption characteristics based on their surface chemistry and functional groups.^108,109^ Modifications using alkali treatments have been shown to enhance the adsorption capacity of HDAC for dyes and heavy metals, indicating that the chemical activation of HDAC can significantly improve its performance in wastewater treatment applications.^85,93,110^

The integration of hydrochar and HDAC into hybrid treatment systems, which may include biological processes or other advanced oxidation methods, presents a promising avenue for enhancing the overall efficacy of pharmaceutical contaminant removal. The synergistic effects of combining hydrochar with other treatment modalities can lead to improved degradation rates and removal efficiencies, thereby addressing the complex nature of pharmaceutical pollutants in wastewater.^57,105^ Furthermore, the potential for hydrochar to act as an electron shuttle in anaerobic digestion processes has been highlighted, suggesting that its application could extend beyond adsorption to include roles in microbial metabolism and nutrient recovery.^111^

### 3.3. Mechanisms of pharmaceutical wastewater adsorption onto hydrochar and HDAC

**Fig. 4:**
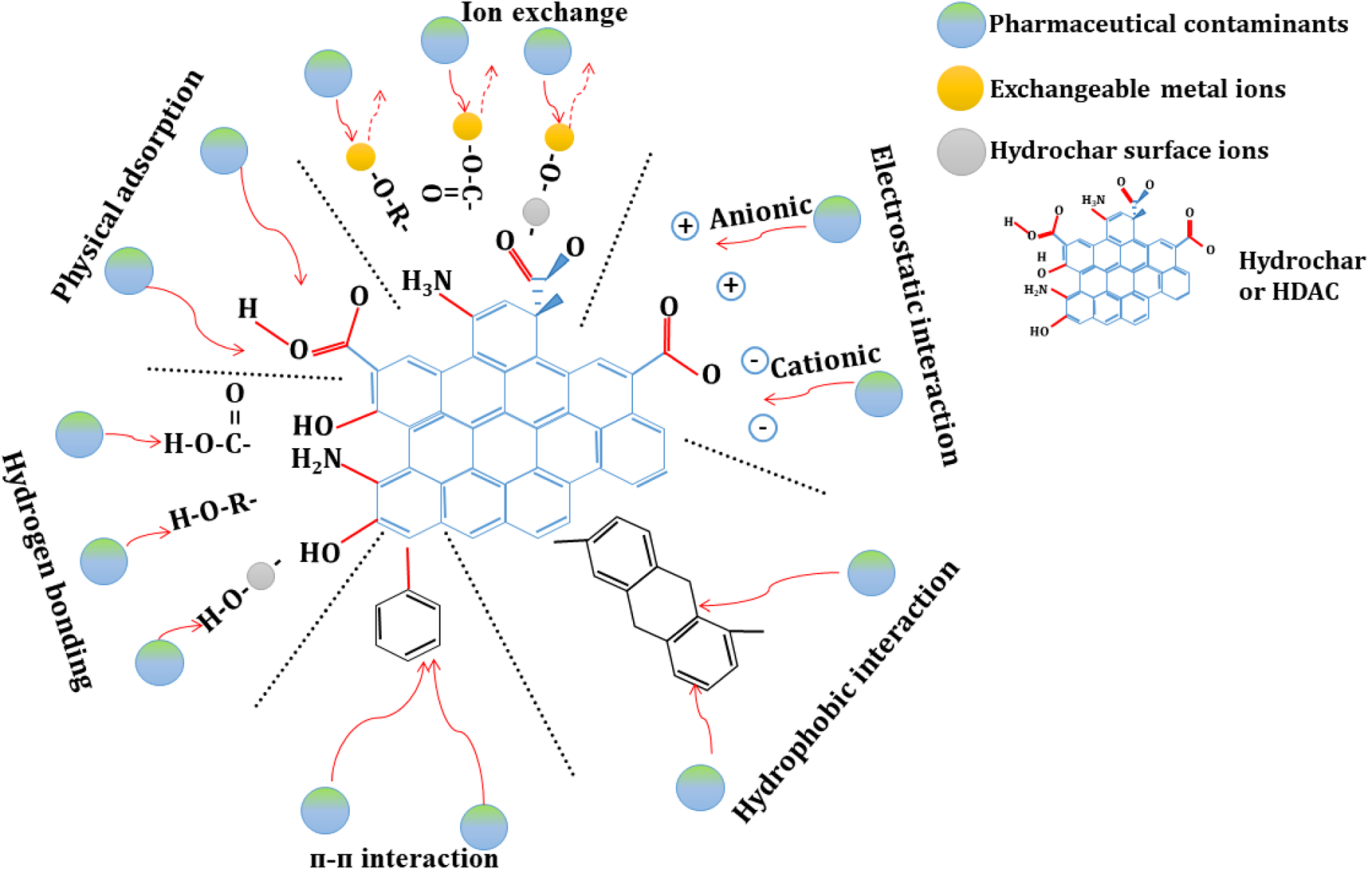
Mechanism of adsorption of pharmaceutical pollutants by hydrochar and HDAC.

The adsorption of pharmaceutical contaminants onto hydrochar and HDAC is governed by a complex interplay of chemical and physical interactions, which are contingent upon both the surface properties of hydrochar or HDAC and specific characteristics of the pollutants. The primary mechanisms underlying this adsorption process encompass hydrogen bonding, π–π interactions, electrostatic interactions, hydrophobic interactions and physical adsorption. The efficacy of adsorption is significantly influenced by the preparation methods employed in synthesizing hydrochar or HDAC and the composition of its functional groups (Fig. 4).

To elucidate the mechanistic superiority of hydrochar or HDAC and reinforce the originality of this review, a deeper analysis of its adsorption mechanisms is warranted, particularly in comparison to biochar and commercial adsorbents. Hydrochar's rich oxygenated functional groups (–OH, –COOH), preserved during hydrothermal carbonization (HTC) at 180–250 °C, enable robust hydrogen bonding and ion exchange interactions with polar pharmaceutical contaminants. For instance, tetracycline (p*K*_a_ 3.4–9.7) and ciprofloxacin (p*K*_a_ 5.9–8.8) exhibit adsorption capacities ranging from 1.1 to 650.8 μg g^−1^, optimized at pH 6–8, where negatively charged or neutral drug molecules are effectively retained (Table 5). This contrasts with biochar, produced *via* pyrolysis at 300–700 °C as reported by Ouyang *et al.* (2020),^27^ which, due to extensive aromatic condensation, possesses fewer oxygenated groups and relies predominantly on π–π interactions with aromatic pharmaceuticals like diclofenac, resulting in lower efficacy for polar compounds. Hydrochar's partial aromatization supports π–π interactions with compounds such as carbamazepine, though less pronounced than biochar. At the same time, its hydrophobicity is significantly enhanced through chemical activation (*e.g.*, ZnCl_2_ activation yielding 1326 m^2^ g^−1^), surpassing non-activated biochar's typical surface area of 500–1000 m^2^ g^−1^.^27^

**Table 5:** Comparative characteristics of hydrochar, biochar and commercial activated carbon for pharmaceutical adsorption

Material	Production method	Surface area (m^2^ g^−1^)	Key functional groups	Main mechanism	Adsorption capacity (μg g^−1^)
Hydrochar (this review)	HTC (180–250 °C)	0.0043–2431	–OH, –COOH	H-bonding, ion exchange	1.1–650.8
Biochar^27^	Pyrolysis (300–700 °C)	500–1000	Aromatic rings	π–π interactions	210–230 (ref. 112)
Commercial AC	High-temp activation	500–1500 ref. 113	Few oxygenated groups	π–π, hydrophobic^114,115^	69–277 (ref. 116 and 117)

A standardized comparative analysis further highlights hydrochar's advantages over commercial activated carbons, which exhibit surface areas of 500–1500 m^2^ g^−1^ but require energy-intensive drying and complex activation processes, often depleting surface functional groups. Table 5 summarizes these differences, demonstrating hydrochar's cost-effectiveness and selective adsorption for antibiotics, driven by its tunable porosity and functional group density. The mechanistic insights, derived from empirical data, establish hydrochar as a highly effective and sustainable solution for mitigating antibiotic pollution – a subject insufficiently addressed in previous reviews – and provide a basis for assessing its efficacy across various wastewater types.

#### 3.3.1. Hydrogen bonding

The adsorption of pharmaceutical contaminants onto hydrochar and HDAC is significantly influenced by hydrogen bonding, particularly for compounds that possess polar functional groups such as hydroxyl (–OH), carboxyl (–COOH), and amine (–NH_2_) groups (Fig. 4). Hydrochars that are enriched with oxygen-containing functional groups, including carboxyl and hydroxyl moieties, provide numerous active sites conducive to forming hydrogen bonds, thereby enhancing the adsorption efficiency of these contaminants. This phenomenon is particularly pronounced for pharmaceuticals characterized by high polarity, making hydrogen bonding a pivotal mechanism in the adsorption of antibiotics and anti-inflammatory drugs.

The role of hydrogen bonding in the adsorption process is well-documented in the literature. For instance, Sun *et al.*(2015)^102^ demonstrated that modifying hydrochars with KOH significantly increased the content of oxygen-containing functional groups, which improved the HDAC's capacity for adsorbing contaminants. This finding underscores the importance of functional group composition in enhancing adsorption capabilities. Similarly, Quintero-Álvarez *et al.* (2022)^118^ observed that the formation of hydrogen bonds between pharmaceutical molecules and the surfaces of metal–organic frameworks (MOFs) was evidenced by changes in the FTIR spectra, indicating that such interactions are crucial for effective adsorption. Furthermore, Soroush *et al.* (2022)^119^ reported that an increase in the water-to-biomass ratio during hydrothermal carbonization resulted in a greater abundance of hydroxyl and carboxyl groups in the resulting hydrochar, which corresponded with enhanced adsorption capacities for contaminants.

#### 3.3.2. π–π interactions

The interaction of pharmaceuticals with hydrochars, particularly those containing graphitic carbon layers or aromatic surfaces, is a significant area of research due to the prevalence of aromatic compounds in wastewater. Aromatic pharmaceuticals, such as fluoroquinolones and anti-inflammatory drugs, exhibit strong interactions with hydrochars through π–π stacking, which is enhanced by the structural characteristics of the hydrochars (Fig. 4). Hydrochars produced at elevated temperatures or subjected to chemical activation show increased aromaticity, which is critical for the effective adsorption of these contaminants.

Research indicates that the graphitization of hydrochars, which occurs at higher carbonization temperatures, leads to the formation of graphitic structures that enhance π–π interactions with aromatic pharmaceuticals. For instance, Qian *et al.* (2018)^120^ demonstrated that higher hydrothermal liquefaction temperatures resulted in a greater degree of graphitization, as evidenced by the decreasing intensity ratio of the D to G bands in Raman spectra, indicating improved structural order and aromaticity. Similarly, Liu *et al.* (2016)^121^ noted that the *in situ* formation of zerovalent iron nanoparticles during hydrochar synthesis contributed to the transition from amorphous to more graphitic carbon structures, further enhancing the material's adsorption capabilities.

The role of chemical activation in increasing the surface area and porosity of HDAC is also critical. Xu *et al.* (2013)^122^ reported that chemical activation with agents like KOH can significantly enhance the surface area of HDAC, reaching values over 2500 m^2^ g^−1^, which is beneficial for adsorption applications. This increased surface area, combined with the improved aromaticity from higher temperature processing, creates a more favorable environment for the adsorption of aromatic pharmaceuticals. Fernández *et al.* (2013)^123^ also highlighted that HDAC exhibits superior adsorption properties for emerging organic contaminants, including pharmaceuticals, due to its tailored surface characteristics. Also, oxygen-containing functional groups, such as carboxyl and hydroxyl, can further influence adsorption. Niu *et al.* (2023)^124^ found that modifications to hydrochars increased these functional groups, which positively correlated with the adsorption capacity for specific pharmaceuticals like sulfamethoxazole and carbamazepine. This suggests that while π–π stacking is a primary interaction mechanism, the presence of functional groups can enhance the overall adsorption process through additional electrostatic interactions.

#### 3.3.3. Hydrophobic interactions

The analysis of hydrochar and HDAC's effectiveness in removing pharmaceutical contaminants from wastewater is grounded in understanding hydrophobic interactions and the physicochemical properties of hydrochar. Hydrochars, particularly those with high aromaticity and reduced oxygen content, exhibit enhanced capabilities for adsorbing non-polar pharmaceutical contaminants such as hormones and hydrophobic antibiotics. This phenomenon is primarily attributed to the hydrophobic interactions that occur when these non-polar contaminants are attracted to the hydrophobic surfaces of hydrochar.

HDAC produced at elevated temperatures or treated with chemical activators like KOH demonstrates significantly improved hydrophobicity. The activation process enhances the surface area and reduces the presence of oxygen-containing functional groups, which can hinder adsorption by increasing hydrophilicity.^78,123^ For instance, KOH treatment has been shown to modify the surface characteristics of HDAC, leading to a decrease in polar functional groups and an increase in hydrophobic sites, thereby facilitating the adsorption of hydrophobic pharmaceuticals.^26,123^ This observation is consistent with findings that demonstrate a direct correlation between decreased surface oxygen content and enhanced hydrophobic interactions, positioning hydrochar as an effective adsorbent for the removal of hydrophobic pharmaceuticals from wastewater.^41,79^ Moreover, the structural characteristics of hydrochar, such as its aromaticity, play a crucial role in its adsorption capacity. Hydrochars with higher aromatic content tend to have stronger hydrophobic interactions with pharmaceutical contaminants, which can be further enhanced by the carbonization process that increases the degree of carbonization and aromaticity.^41,125^ Studies have shown that the adsorption mechanisms depend not only on hydrophobic interactions but also on other forces, including π–π stacking and van der Waals interactions, which play a significant role in the adsorption of complex pharmaceutical compounds.^126^

#### 3.3.4. Physical adsorption

Physical adsorption is characterized by its non-specific nature, which contrasts with the more selective chemical interactions. Despite this, it plays a significant role in the adsorption of antibiotics, particularly those that are large and structurally complex, such as tetracycline, amoxicillin and ciprofloxacin. The effectiveness of hydrochar as an adsorbent for these antibiotics is largely contingent upon its surface area and pore structure, which facilitate the diffusion of contaminants into the micro- and mesoporous networks of the hydrochar.^80,102^ Research indicates that antibiotics with larger molecular sizes benefit from the physical adsorption process, as they require sufficient pore sizes for effective diffusion. For instance, tetracycline, which has a molecular size of approximately 1.5 nm, can be effectively adsorbed by hydrochars with optimized pore structures that allow easy access to the adsorbent's internal surfaces.^93,127^ Enhancing HDAC's porosity through activation techniques, such as KOH or CO_2_ activation, has significantly improved its adsorption capacity for these contaminants.^86,104^

The surface area and pore structure of hydrochar are critical factors that determine its adsorption capacity. Hydrochars produced from various feedstocks exhibit different physicochemical properties, influencing their ability to adsorb antibiotics. For example, hydrochars derived from agricultural residues often possess higher surface areas and more developed pore structures than those derived from municipal waste.^103^ This variability underscores the importance of selecting appropriate feedstocks and activation methods to optimize HDAC for specific applications in antibiotic removal. Studies have demonstrated that the specific surface area of HDAC can be significantly increased through chemical activation, resulting in a greater number of available adsorption sites. For instance, KOH activation has been reported to enhance the surface area of HDAC from 20 m^2^ g^−1^ to over 300 m^2^ g^−1^, thereby improving its capacity to adsorb antibiotics such as amoxicillin and ciprofloxacin.^85,128^ Functional groups, such as hydroxyl and carboxyl groups, further contribute to the adsorption process by providing additional binding sites for antibiotic molecules.

#### 3.3.5. Surface complexation

The adsorption of pharmaceutical contaminants, particularly those with polar functional groups or metal ions, is significantly influenced by surface complexation mechanisms in hydrochar. This process involves the formation of chemical complexes between the active functional groups on the hydrochar surface and the contaminant molecules, which enhances the removal efficiency of pharmaceuticals that exhibit a high affinity for such interactions. Hydrochar typically contains a diverse array of oxygen- and nitrogen-containing functional groups, including –COOH, –OH and –NH_2_ groups. These functional groups can act as electron donors or acceptors, facilitating the formation of coordination bonds with pharmaceutical contaminants.^48,76^

Surface complexation is a critical mechanism for the adsorption of pharmaceuticals, particularly those that are polar or ionic. Functional groups on the hydrochar surface enhance their ability to interact with pharmaceutical molecules through various bonding mechanisms. For instance, carboxyl and hydroxyl groups can form hydrogen bonds with polar pharmaceutical compounds, while amine groups can engage in ionic interactions with negatively charged contaminants.^129,130^ This chemical affinity is particularly relevant for antibiotics such as tetracycline and amoxicillin, which possess polar functional groups that readily interact with the hydrochar surface.^131,132^ Research has demonstrated that the effectiveness of hydrochar in removing pharmaceutical contaminants is closely linked to its surface chemistry. For example, introducing oxygen-containing functional groups through chemical activation has been shown to significantly enhance the adsorption capacity of HDAC for various pharmaceuticals.^133,134^ The increased presence of these functional groups improves the hydrochar and HDAC's ability to form complexes with contaminants and enhances its overall adsorption kinetics and isotherm characteristics.^122,135^

#### 3.3.6. Ion exchange

Ion exchange is a vital mechanism for the adsorption of charged pharmaceutical molecules and metal ions. Hydrochars rich in surface functionalities, such as –COOH, –OH and phenolic groups, are particularly effective in facilitating this process. These functional groups can release hydrogen ions (H^+^) or other cations, such as sodium (Na^+^) or potassium (K^+^), in exchange for positively charged contaminants in the solution.^102,119^ For instance, carboxyl and phenolic groups enhance the hydrochar's ability to interact with cationic pharmaceuticals, such as amoxicillin and tetracycline, which possess polar functional groups that can readily engage in ion exchange reactions.^103^

In numerous instances, the adsorption of pharmaceuticals is governed by the interplay of multiple mechanisms. For example, hydrochar derived from spent coffee grounds employs hydrogen bonding and electrostatic interactions to adsorb sulfamethoxazole and sulfadiazine effectively. Similarly, hydrochar derived from acid-treated rice husks facilitates hydrogen bonding, electrostatic interactions, and hydrophobic interactions, leading to improved adsorption of norfloxacin, with a maximum capacity of 51.86 mg g^−1^.^62^ The synergistic nature of these mechanisms significantly augments the versatility and efficacy of hydrochar in treating pharmaceutical wastewater. Furthermore, surface complexation and ion exchange frequently work with other mechanisms, such as hydrogen bonding and electrostatic interactions, to improve adsorption efficiency. For instance, in sulfamethoxazole adsorption, surface complexation involving the carboxyl groups present on the hydrochar and the sulfonamide functional groups is augmented by hydrogen bonding, culminating in superior removal efficiency.^54^ Likewise, in HDAC treated with ZnCl_2_, the synergistic effects of ion exchange, π–π interactions, and chemisorption yield remarkable adsorption performance for ciprofloxacin.

The optimization of adsorption mechanisms can be achieved through chemical activation and surface modification to enrich functional groups. Acid treatments, such as those employing citric acid or hydrochloric acid, facilitate the introduction of additional carboxyl groups, enhancing ion exchange and surface complexation capacities. Furthermore, adjusting the pH to correspond with the ionization state of the contaminants is crucial for maximizing adsorption efficiency, particularly for pharmaceuticals that exhibit pH-dependent behavior. The efficacy of hydrochar in adsorbing pharmaceutical contaminants is attributable to its multifaceted interaction mechanisms, which can be tailored through careful selection of feedstock, processing conditions, and activation techniques. Hydrogen bonding and electrostatic interactions are predominant for polar and ionic pharmaceuticals, while π–π and hydrophobic interactions are essential for the adsorption of non-polar and aromatic compounds. Although physical adsorption is generally less specific, it is complementary by augmenting adsorption capacity by providing high surface area and pore volume. Future advancements in the design of hydrochar and HDAC should prioritize the optimization of these mechanisms through functionalization and hybrid applications, thereby addressing the diverse challenges associated with pharmaceutical wastewater treatment.

## 4. Challenges and future directions

Hydrochar and HDAC have demonstrated considerable promise as a sustainable adsorbent for removing pharmaceutical contaminants from wastewater. Nevertheless, the broader implementation of hydrochar and HDAC encounters several challenges that necessitate innovative solutions. While these challenges are substantial, they offer opportunities for future advancements in hydrochar technology.

### 4.1. Challenges

One of the primary limitations of hydrochar is its relatively low adsorption capacity, particularly concerning highly persistent pharmaceutical compounds. Hydrochars produced without applying advanced activation techniques typically exhibit low surface areas and minimal porosity, which significantly constrains their effectiveness as adsorbents. For example, hydrochars derived from horse manure and tomato waste demonstrate Brunauer–Emmett–Teller (BET) surface areas of merely 4.62 m^2^ g^−1^ and 0.74 m^2^ g^−1^, respectively. Consequently, these low surface area values correlate with limited adsorption capacities for pollutants such as ciprofloxacin, which are recorded at 1.8 μg g^−1^ and 1.5 μg g^−1^ for horse manure and tomato waste hydrochars, respectively. This inherent limitation restricts the efficacy of hydrochar in treating wastewater characterized by high concentrations of contaminants or complex matrices containing multiple pollutants.

Another significant challenge of using hydrochar and HDAC as an adsorbent is the non-selective adsorption characteristics. Hydrochar tends to adsorb a broad spectrum of contaminants, including non-target compounds, which can lead to diminished efficiency in removing specific pharmaceuticals. In wastewater characterized by a mixture of contaminants, competitive adsorption phenomena can impede the uptake of priority pollutants, such as antibiotics or anti-inflammatory drugs. This competition among various contaminants ultimately reduces the overall treatment performance of hydrochar in wastewater applications. The non-selective nature of hydrochar and HDAC adsorption poses a critical barrier to its effectiveness, particularly in complex wastewater matrices where the presence of multiple pollutants can significantly alter adsorption dynamics. As a result, the ability of hydrochar and HDAC to target and remove specific pharmaceutical contaminants is compromised, necessitating further research into strategies that could enhance its selectivity and adsorption efficiency.

Environmental and operational factors significantly influence the adsorption efficiency of hydrochar and HDAC, presenting additional challenges to its application in wastewater treatment. Key factors such as pH, ionic strength, and the presence of natural organic matter (NOM) can markedly affect the adsorption process and, consequently, the overall effectiveness of hydrochar and HDAC as an adsorbent. For instance, pH variations can alter the ionization state of pharmaceutical compounds and the surface charge of hydrochar. These changes can impact critical adsorption mechanisms, including hydrogen bonding and electrostatic interactions. A shift in pH may enhance or inhibit the adsorption of specific pharmaceuticals, thereby affecting the overall removal efficiency. Moreover, high salinity or NOM in wastewater can obstruct active sites on the hydrochar and HDAC surfaces, further diminishing their adsorption capacity. High ionic strength can lead to the compression of the electrical double layer surrounding the hydrochar particles, which may reduce the electrostatic attraction between hydrochar and HDAC and charged pharmaceutical molecules. Similarly, NOM can compete with pharmaceuticals for adsorption sites, limiting active sites' availability for target contaminants. These environmental and operational factors underscore the complexity of using hydrochar and HDAC in real-world wastewater treatment scenarios, necessitating further research to optimize conditions for enhanced adsorption performance.

Scaling up hydrochar and HDAC production to meet industrial demand presents a significant challenge. Although HTC is recognized for its energy efficiency compared to pyrolysis, the transition to large-scale production is impeded by various cost barriers. Key factors contributing to these increased operational costs include the pre-treatment of biomass feedstocks, the utilization of chemical activation agents such as KOH or ZnCl_2_ and the management of by-products generated during the HTC process. Pre-treatment of biomass is essential to enhance the quality and yield of hydrochar; however, it often involves additional processing steps that can be costly and time-consuming. Furthermore, incorporating chemical activation agents, which are necessary to improve the surface area and porosity of hydrochar, adds to the overall expense of production. Managing by-products from the HTC process also poses logistical and financial challenges, as effective disposal or utilization strategies must be developed to mitigate environmental impacts. In addition to production costs, the regeneration of spent hydrochar remains a formidable challenge. Current regeneration methods, including thermal or chemical desorption, are frequently energy-intensive and can compromise the structural integrity of the hydrochar. This degradation limits the reusability of hydrochar and HDAC, thereby reducing their economic viability as a sustainable adsorbent in wastewater treatment applications. Addressing these challenges is crucial for successfully scaling hydrochar and HDAC production to meet the demands of industrial applications. Future research should focus on optimizing production processes, reducing costs, and developing efficient regeneration techniques to enhance the sustainability and applicability of hydrochar and HDAC.

### 4.2. Future directions

To address the challenges associated with hydrochar and HDAC utilization, future research and development efforts should concentrate on several promising strategies. A pivotal area of focus is enhancing activation techniques to improve the adsorption capacity of hydrochar and HDAC. Chemical activation methods, particularly those employing agents such as KOH or ZnCl_2_, have shown considerable efficacy in augmenting the surface area and adsorption capabilities of HDAC. For instance, HDAC treated with ZnCl_2_ has achieved a Brunauer–Emmett–Teller (BET) surface area of 1326 m^2^ g^−1^, indicating a significant enhancement in its adsorption potential.^78^ Furthermore, incorporating green activation methods, such as steam treatment or bio-based catalysts, presents an opportunity to reduce production costs and mitigate environmental impacts while simultaneously improving the performance of hydrochar. In addition to chemical activation, the structural and compositional modifications of HDAC can further enhance its adsorption properties. The introduction of oxygenated functional groups through various modification processes has been shown to significantly influence the adsorption capacity of hydrochar for multiple contaminants, particularly positively charged species. The presence of these functional groups facilitates interactions such as ion exchange and hydrogen bonding, which are crucial for effective adsorption. Moreover, optimizing HTC parameters, including the water-to-biomass ratio, can produce hydrochars with improved porosity and functional group density, enhancing their adsorption capacity.

To address the specific bottlenecks of low adsorption capacity and pH sensitivity, future research should prioritize green activation methods, such as H_2_O_2_/HCl or citric acid, which have demonstrated the ability to increase SSA to 100–500 m^2^ g^−1^ and enhance adsorption capacities for sulfamethoxazole and ciprofloxacin without requiring high-energy inputs. For instance, H_2_O_2_/HCl activation of orange peel HDAC increases oxygenated functional groups, improving hydrogen bonding and reducing pH dependency (optimal at pH 6–8), while also lowering costs compared to KOH or ZnC_2_ activation. Morover, optimizing hydrothermal carbonization (HTC) with acid/alkali treatments (*e.g.*, 180–240 °C, 20 h) can increase pore volume to 0.3–0.4 cm^3^ g^−1^, mitigating sensitivity to pharmaceutical compounds by enhancing selectivity through inner-sphere complexation.

A significant avenue for future research is the functionalization of hydrochar and HDAC to enhance their selectivity for specific pharmaceutical compounds. By incorporating various functional groups, such as amino, sulfonic or thiol groups, hydrochar and HDAC can be engineered to improve their adsorption efficiency for targeted pollutants. For instance, introducing functional groups that facilitate hydrogen bonding or π–π interactions may enhance the adsorption capacity for antibiotics. In contrast, the addition of negatively charged groups could promote stronger electrostatic interactions with cationic beta-blockers. The presence of oxygen-containing functional groups on the surface of hydrochar and HDAC has been shown to play a crucial role in its adsorption capabilities. These groups not only enhance the interaction with organic pollutants but also contribute to the overall hydrophilicity of the hydrochar and HDAC, which is essential for effective adsorption processes.

To mitigate non-selective adsorption, targeted functionalization strategies, such as incorporating metal–organic frameworks (MOFs, *e.g.*, ZIF-8) onto magnetic pine sawdust HDAC, have achieved >90% reusability for tetracycline through selective chemisorption and π–π interactions. Similarly, steam-activated grape stalk hydrochar enhances selectivity for diclofenac *via* pore-filling mechanisms. Promising approaches include introducing thiol or amino groups for antibiotics (*e.g.*, sulfamethoxazole) or negatively charged sulfonic groups for cationic beta-blockers, combined with hybrid systems like magnetic Fe_3_O_4_-HDAC to minimize competitive adsorption.

Developing composite materials that integrate hydrochar with advanced materials such as graphene or metal–organic frameworks represents a promising strategy for enhancing adsorption performance. By creating hybrid materials, the functional groups in hydrochar can be effectively combined with these advanced materials' high surface area and porosity characteristics. This leads to improved adsorption capabilities for a diverse array of contaminants. The incorporation of graphene into hydrochar composites is particularly noteworthy due to graphene's exceptional properties, including its high specific surface area and mechanical strength. These attributes can significantly enhance the adsorption performance of hydrochar, particularly for organic pollutants. For instance, studies have demonstrated that combining hydrochar with graphene oxide can yield materials with superior adsorption characteristics, facilitating the removal of contaminants such as heavy metals and pharmaceuticals from aqueous solutions. The synergistic effects arising from this integration can lead to enhanced interactions between the adsorbent and the target pollutants, thereby improving overall efficiency. Moreover, the functionalization of HDAC by incorporating metal–organic frameworks can further augment its adsorption capabilities. Metal–organic frameworks are known for their tunable porosity and high surface area, which can complement the properties of hydrochar and create a more effective adsorbent material. Combining hydrochar's inherent functional groups with the structural advantages of metal–organic frameworks can facilitate stronger interactions with a broader range of contaminants, enhancing selectivity and efficiency in adsorption processes.

To overcome bottlenecks in scaling up and regeneration inefficiency, developing composites such as graphene-hydrochar or MOF-functionalized materials can reduce pre-treatment costs by utilizing waste feedstocks (*e.g.*, spent coffee grounds, rice husks) within a circular economy framework. For regeneration, microwave desorption combined with advanced oxidation processes (AOPs) achieves >85% recovery after five cycles, minimizing energy-intensive thermal methods while preserving structural integrity. Pilot-scale HTC with waste heat recovery from industrial by-products can reduce operational costs by 20–30%, while treating aqueous phase by-products through anaerobic digestion. Life cycle assessments (LCA) should be integrated to evaluate economic viability, such as the application of magnetic sewage sludge HDAC in real wastewater treatment facilities.

Optimizing the HTC process is essential for enhancing the properties of hydrochar. Modifications to key parameters, including temperature, residence time and biomass-to-water ratio, are critical in producing HDAC with improved surface characteristics tailored to specific pollutant removal applications. For example, elevated temperatures facilitate increased carbonization and enhance the density of functional groups, thereby improving the adsorption capacity for hydrophobic compounds. Moreover, HTC processes incorporating acid or alkali treatment can significantly improve the porosity and functionalization of the resulting HDAC.

The integration of hydrochar and HDAC production within a circular economy framework offers significant opportunities for sustainable development. Utilizing agricultural and industrial waste as feedstocks, hydrochar production facilitates waste valorization while promoting sustainable resource management. Materials such as spent coffee grounds, rice husks, and grape seeds are cost-effective raw inputs, aligning with eco-friendly practices by reducing waste and repurposing biomass into value-added products.

Efforts should prioritize the development of efficient regeneration technologies to improve the reusability of hydrochar. Low-energy regeneration methods, such as microwave or ultrasonic desorption, are up-and-coming as they help maintain the structural integrity of hydrochar and HDAC while minimizing operational costs. Also, integrating adsorption with advanced oxidation processes offers a dual benefit by enabling simultaneous regeneration of hydrochar and degradation of adsorbed contaminants, thereby enhancing overall process efficiency.

Field studies and real-world applications are crucial for evaluating the performance of hydrochar under practical operating conditions. Although laboratory studies offer essential insights, wastewater treatment facilities often encounter complex matrices and fluctuating operational parameters that may not be fully replicated in controlled environments. Field-scale experiments and life cycle assessments can generate essential data on the feasibility, environmental impact, and economic viability of hydrochar and HDAC applications in real-world scenarios. This comprehensive approach ensures that hydrochar technologies are both practical and sustainable at scale.

Policy and regulatory support will be pivotal in driving the widespread adoption of hydrochar technologies. Incentives promoting sustainable adsorbents, establishing clear guidelines for pharmaceutical effluent management, and providing funding and support for research and innovation in water treatment technologies can foster an enabling environment for large-scale hydrochar deployment. Such measures will enhance the practicality of hydrochar applications and contribute to broader sustainability goals in wastewater management.

In summary, these recommendations offer a coherent roadmap: initiate lab-scale functionalization (*e.g.*, MOFs for selectivity), transition to pilot-scale with green activation methods (H_2_O_2_/citric acid to reduce costs), and support policy through incentives for R&D (*e.g.*, funding for field trials), addressing bottlenecks within the next 5–10 years.

## 5. Conclusion

Hydrochar and HDAC have exhibited substantial efficacy as an adsorbent for removing pharmaceutical contaminants from wastewater, with adsorption capacities influenced by the preparation methods and the specific types of pollutants involved. For instance, hydrochar produced from horse manure through straightforward hydrothermal carbonization achieved adsorption capacities of approximately 1.8 μg g^−1^ for ciprofloxacin and 1.1 μg g^−1^ for sulfamethoxazole, primarily facilitated by hydrophobic interactions and hydrogen bonding. In contrast, the application of more sophisticated activation techniques, such as ZnCl_2_ activation, resulted in HDAC with significantly enhanced surface areas (up to 1326 m^2^ g^−1^) and adsorption capacities of 416.7 μg g^−1^ for ciprofloxacin, thereby underscoring the potential benefits of tailored activation processes.

The adaptability of HDAC is further evidenced by its capacity to be derived from various feedstocks, including agricultural residues (*e.g.*, rice husks and grape seeds) and industrial by-products (*e.g.*, spent coffee grounds). For example, hydrochar synthesized from grape seeds and activated with potassium hydroxide (KOH) demonstrated an impressive adsorption capacity of 650.8 mg g^−1^ for sulfamethoxazole, highlighting the significance of enhanced porosity and the availability of functional groups in optimizing adsorption performance. Moreover, the sustainable production process of hydrochar, which utilizes biomass waste and operates at relatively low temperatures, contributes to a reduced environmental footprint compared to conventional adsorbents such as activated carbon.

Despite the promising attributes of hydrochar and HDAC, several challenges warrant critical attention to ensure its practical scalability and long-term viability in wastewater treatment. Notably, non-selective adsorption remains a significant limitation, as hydrochar's broad affinity for various contaminants can reduce its efficiency in targeting specific pharmaceuticals in complex wastewater matrices. This lack of selectivity often leads to competitive adsorption, where co-existing pollutants diminish the material's effectiveness for priority contaminants. Moreover, hydrochar and HDAC's sensitivity to pH variations poses a considerable challenge, as adsorption mechanisms, such as π–π stacking and electrostatic interactions, are highly pH-dependent. For instance, the adsorption behavior of levofloxacin fluctuates significantly under different pH conditions, with optimal performance typically observed at neutral pH, complicating its application in diverse wastewater environments. Furthermore, inefficient regeneration processes hinder hydrochar's reusability, a critical factor for cost-effectiveness and sustainability. Current regeneration methods often fail to fully restore adsorption capacity or require energy-intensive processes, undermining hydrochar's environmental benefits. These challenges highlight the need for targeted research to develop selective hydrochar materials, pH-robust adsorption mechanisms and low-energy regeneration techniques to enhance their practical utility.

Addressing these challenges will be pivotal for scaling hydrochar and HDAC's application in sustainable wastewater management. Future research should optimize activation methods to enhance selectivity, explore surface modifications to stabilize performance across pH ranges and develop innovative regeneration technologies, such as microwave or ultrasonic desorption, to improve reusability while maintaining structural integrity. By overcoming these hurdles, hydrochar and HDAC can solidify their role as a versatile and eco-friendly adsorbent, significantly mitigating pharmaceutical pollutants' environmental impact. These variations reflect discrepancies arising from feedstock composition and activation methods: lignin-rich residues, such as rice husks, achieve a SSA of up to 1326 m^2^ g^−1^ following ZnCl_2_ activation, enhancing chemisorption, whereas low-carbon feedstocks like horse manure limit capacity due to pore collapse. Compared to traditional activated carbon, hydrochar offers a reduced environmental footprint through low-temperature hydrothermal carbonization (HTC, 180–250 °C) and effective utilization of wet biomass.

## Author contributions

Thi Mai Vu and Ngoc Thuan Le: data collection; Huu-Tap Van, Thi Minh Phuong Nguyen, Dinh-Trinh Tran: writing draft, review and revised manuscript.

## Conflicts of interest

The authors have not disclosed any competing interests.

## Data Availability

Data associated with this study have not been deposited into a publicly available repository. Data will be made available on request.
